# Abnormal Ca^2+^ handling and reduced I_to_ contribute to citalopram-induced QT prolongation and cardiac arrhythmias

**DOI:** 10.3389/fphar.2025.1613438

**Published:** 2025-09-04

**Authors:** Yangpeng Li, Jiamin Xie, Yuqing Zheng, Jianhong Li, Min Zhao, Li Liu, Hongping Shen, Ming Lei, Xiaoqiu Tan, Juan Xiao, Xueru Liu, Tangting Chen

**Affiliations:** ^1^ Key Laboratory of Medical Electrophysiology of the Ministry of Education, Institute of Cardiovascular Research, Southwest Medical University, Luzhou, China; ^2^ Department of Cardiology, Affiliated Hospital, Southwest Medical University, Luzhou, China; ^3^ Department of Anesthesiology, Affiliated Hospital, Southwest Medical University, Luzhou, China; ^4^ The Affiliated Traditional Chinese Medicine Hospital, Southwest Medical University, Luzhou, China; ^5^ Department of Pharmacology, University of Oxford, Oxford, United Kingdom

**Keywords:** citalopram, selective serotonin reuptake inhibitor, cardiac arrhythmia, action potential, alternans

## Abstract

**Background:**

Citalopram (CIT) is widely used as an anti-depressant and has been reported to be associated with QT interval prolongation and increased vulnerability of torsades de pointes (TdP), although the underlying mechanism remains unclear.

**Objective:**

The aim of this study is to determine the proarrhythmic properties and underlying mechanisms of CIT.

**Methods:**

Mice were intraperitoneally injected with CIT or saline (SAL) for 4 weeks. Echocardiograms and electrocardiograms were performed to evaluate the cardiac electrophysiological and hemodynamic properties. Proarrhythmic mechanisms of CIT were then explored using optical mapping. Finally, transcriptomic array, RT-qPCR, and whole-cell patch-clamp were conducted to explore and validate the potential ionic mechanisms of CIT-related electrical abnormalities.

**Results:**

CIT treatment induced QT prolongation and increased the vulnerability of cardiac arrhythmias in the absence of structural or hemodynamic changes. Optical mapping showed that action potential duration (APD) and Ca^2+^ transient duration (CaTD) were prolonged, but the degree of prolongation was heterogeneous, resulting in impaired Vm–Ca^2+^ coupling in CIT-treated hearts. Meanwhile, CaT alternans exhibited a regional preference for initiating ventricular tachyarrhythmias in hearts treated with CIT. The transcriptomic array showed that multiple potassium channels were downregulated in hearts treated with CIT, which were confirmed by RT-qPCR. Prolonged APD and downregulated transient outward potassium current (I_to_) and L-type calcium current (I_Ca-L_) were recorded in isolated single cardiomyocytes with the patch-clamp technique after CIT treatment.

**Conclusion:**

CIT treatment resulted in QT prolongation and higher susceptibility of ventricular arrhythmia. Regions with serious cardiac alternans were mainly responsible for initiation of CIT-related arrhythmias. Abnormal Ca^2+^ handling and decreased I_to_-related gene expression likely underlie the ionic mechanism and may be a novel target for CIT-related arrhythmias.

## 1 Introduction

Citalopram (CIT) is a selective serotonin reuptake inhibitor (SSRI), which is widely used as an anti-depressant in clinical practice (Cooke and Waring, 2013). Despite its promising therapeutic effects, there is accumulating evidence suggesting its cardiac side effects, including QT interval prolongation and increased incidence of cardiac arrhythmias (Cooke and Waring, 2013; Tampi et al., 2015; Beach et al., 2018). In September 2011, the Food and Drug Administration (FDA) announced a bulletin restricting the dosage of citalopram due to its adverse effects on electrocardiogram (ECG) abnormalities among elderly people and patients at risk of prolonged QT interval (Vieweg et al., 2012). Some clinical cases have also reported its potential risk of increasing the vulnerability of torsades de pointes (TdP) (Ryan et al., 2022). Meanwhile, CIT had a detrimental effect of causing cardiac remodeling after myocardial infarction (Frey et al., 2016). Theoretical and experimental studies have revealed that patients with long QT syndrome have an increased risk of fatal ventricular tachyarrhythmias (VT), most commonly manifested as TdP. Drug-induced QT prolongation is closely associated with I_ks_ and I_kr_ (Roden, 2019). Of all the SSRI class of drugs, CIT has been confirmed to prolong the QT interval most significantly and has been reported to inhibit activity of multiple ion channels or currents related to repolarization (Beach et al., 2018), especially I_Ks_ and I_Kr_ (Rohrbacher et al., 2012), which is consistent with the mechanism of drug-induced QT prolongation. In addition, some studies on the toxicity of CIT have referred to Nav 1.5 and I_Ca,_ (Witchel et al., 2002; Nakatani and Amano, 2021). Moreover, a recent study on human-induced pluripotent stem cell-derived cardiomyocytes (hiPSC-CMs) indicated that therapeutic concentrations of CIT significantly increased action potential triangulation and proarrhythmic potential but did not explain the underlying ionic mechanism (Faraj et al., 2023). Based on these findings, some drugs have been proven to have a therapeutic effect for CIT-related arrhythmias, such as charcoal and salidroside (Friberg et al., 2006; Zhang et al., 2024). Nevertheless, despite over 2 decades of studies on the cardiac toxicity of CIT, considering current obscure details and evidence, the underlying proarrhythmic mechanism of CIT remains poorly understood. Recent research studies have also revealed that intracellular calcium handling abnormalities play a critical role in arrhythmogenesis (Liao et al., 2021; Qu and Weiss, 2023). However, related studies have rarely been implemented. Here, we performed dual-voltage and calcium optical mapping of Langendorff-perfused hearts and whole-cell patch-clamp to evaluate the electrophysiological properties and proarrhythmic mechanism of high-dose treatment of CIT.

## 2 Materials and methods

### 2.1 Animals

C57BL/6N mice (10–12 weeks of age, no gender restrictions) were provided by the Experimental Animal Center of Southwest Medical University and maintained in a specific pathogen-free facility with *ad libitum* access to food and water. All procedures were approved by the Institutional Animal Ethics Committee at Southwest Medical University, Luzhou, China (No: 20221108-010), which conforms to the Animal Research: Reporting of *In Vivo* Experiments (ARRIVE) guidelines.

### 2.2 Citalopram treatment

CIT (Cat. No: 59729-32-7, MedChemExpress, New Jersey, United States) was administered intraperitoneally as a solution at a high therapeutic concentration of 40 mg/mL (dissolved in PBS), at a dosage of 9.6 mg/kg/day for 4 weeks. The dosage and treatment protocol were based on a published report and were confirmed to have a plasma concentration of over 40 μg/L (Honig et al., 2009).

### 2.3 Echocardiogram and *in vivo* electrocardiogram monitoring

Mice were first anesthetized with 3%–5% isoflurane, maintained on 1%–1.5% isoflurane, and then attached with four leads for baseline ECG monitoring (M150, Biopac, Goleta, California, United States) (heart rate was maintained at 400–500 bpm). ECG recordings were examined at a stable state for over 5 min. The QT interval, PR interval, and RR interval were measured and analyzed. Abnormal cardiac events, such as premature ventricular beats (PVCs), were also recorded. For echocardiogram measurement, mice were anesthetized with 1%–1.5% isoflurane, and then parasternal short-axis section (PSAX) B mode and M mode (Vevo 3100, FUJIFLIM VisualSonics Inc., Canada) were used to evaluate potential changes in hemodynamic and structural remodeling. Primary indicators of cardiac function, such as ejection fraction (EF), fractional shortening (FS), end-systole (s) and end-diastole (d) of the intraventricular septal thickness (IVSs and IVSd), end-systole and end-diastole of left ventricular posterior wall thickness (LVPWs and LVPWd), and end-diastolic and end-systolic left ventricular dimensions (LVIDs and LVIDd), were measured and quantified.

### 2.4 Langendorff-perfused hearts and optical mapping procedures

The detailed procedures of optical mapping have been thoroughly described previously (Li et al., 2023b). In brief, hearts were quickly extracted and mounted on the Langendorff perfusion system. Excitation–contraction uncoupler and optical dyes were perfused in order. Programmed electrical pacing was then performed to induce cardiac alternans and arrhythmias. After acquiring optical signals, basic electrophysiological parameters such as action potential duration (APD), calcium transient duration (CaTD) and their spatial heterogeneity, conduction velocity (CV), and cardiac alternans with reentrant circuits were quantified and analyzed.

### 2.5 Optical mapping pacing protocol

When the heart reached a stable state, it was first paced at a sequence of 100 ms, and then the pacing cycle length was decreased by 10 ms until a pacing cycle length of 50 ms was reached. After testing frequency-dependent alternans and restitution properties, consecutive 50 Hz burst pacing was conducted to induce ventricular arrhythmias (Supplementary Figure S1). This procedure was repeated after ISO (final working concentration 1 µM) intervention.

### 2.6 Details of optical mapping procedures

The detailed protocol refers to our previous publication (Li et al., 2023b). After intraperitoneal heparin treatment and anesthesia with 3% isoflurane, the hearts were quickly isolated and perfused with Krebs solution in NaCl 119, NaHCO_3_ 25, NaH_2_PO_4_ 1.0, KCl 4.7, MgCl_2_ 1.05, CaCl_2_ 1.8, and glucose 10 (mM); the pH was adjusted to 7.35–7.45 with NaOH, and the baths were oxygenated with 95% O_2_ and 5% CO_2_ to maintain the stability of pH. After the Langendorff-perfused hearts reached a stable state, as confirmed by real-time ECG monitoring, 50 µL of blebbistatin stock solution was diluted in 50 mL of circulating Krebs solution (final concentration 10 µM) to minimize contraction artifacts. Then, 15 µL stock solution of Rhod2-AM (final concentration 0.356 mM, 1 mg/mL, stock solution, Cat: ab142780, Abcam, Cambridge, United Kingdom) and 15 µL Pluronic F127 (20% w/v in DMSO, Cat: P3000MP, Invitrogen, California, United States) were diluted in 20 mL Krebs solution and circulated in a total volume of 50 mL for 15 min; next, 10 µL of the stock solution of RH237 (1 mg/mL, final concentration 0.603 mM; stock solution Cat: S1109, Thermo Fisher Scientific, Waltham, Massachusetts, United States) was perfused to obtain simultaneous membrane potential (Vm) and intracellular Ca^2+^ recordings. Once dye loading was accomplished, a custom-designed optical mapping system, including an electron-multiplying charge-coupled device (EMCCD) camera (Evolve 512, Photometrics, Tucson, Arizona, United States) and two light-emitting diodes (LEDs) of 530 nm, were used for excitation of the Ca^2+^ transient and transmembrane voltage probes. CaT fluorescence was acquired using a 585-/40-nm bandpass filter, while Vm fluorescence was collected using a 662-nm long-pass filter. The high-speed EMCCD camera enabled high temporal resolution with a sampling rate of up to 1,000 frames/s and a minimum spatial resolution at the sample of 32 × 32 µm per pixel. The programmed stimulation and ECG recordings were simultaneously controlled using Spik2 software (Cambridge Electronic Design limited, Cambridge, United Kingdom). For optical mapping signal analysis, data were semi-automatically processed using ElectroMap (University of Birmingham, Birmingham, United Kingdom) and OmapScope5 software (MappingLab, Manchester, United Kingdom).

### 2.7 Transcriptomics array

The transcriptomics array was performed at Novogene Co. Ltd. (Beijing, China), in accordance with the manufacturer’s protocols. Total RNA was obtained, and its integrity was evaluated using the RNA Nano 6000 Assay Kit of the Bio-analyzer 2100 system (Agilent Technologies, Clara, California, United States). Subsequently, first-strand cDNA was synthesized, and a library was constructed for transcriptome sequencing. The index-coded samples underwent clustering, and the library preparations were sequenced on an Illumina NovaSeq platform, generating 150 base-pair paired-end reads. The resulting data were subjected to Gene Ontology (GO) and Kyoto Encyclopedia of Genes and Genomes (KEGG) enrichment analyses.

### 2.8 RT-qPCR

Total RNA was extracted from cardiac tissue using the RNA Easy Fast Tissue/Cell Kit (cat. DP451; Tiangen Biotech, Shanghai, China). Following this, the total RNA was subjected to reverse transcription to synthesize cDNA using ReverTra® Ace qPCR RT Master Mix (cat. FSQ-201; Toyobo Life Science, Osaka, Japan). qPCR was conducted using the Taq Pro Universal SYBR qPCR Master Mix (Vazyme Biotech, Nanjing, China) under the following thermal cycling conditions: 95°C for 10 s, 60°C for 25 s, and 72°C for 30 s for 40 cycles. The mRNA expression levels of the target genes were normalized to GAPDH using the 2^−ΔΔCt^ method (Livak and Schmittgen, 2001). Primers used in this study are displayed in Table 1.

**TABLE 1:** Primers for RT-qPCR of different genes.

Gene	Primer sequence (5′→3′)
KCNIP2	F: ACAGACCAAGTTCACACGCA R: CCCCGAAGAATCACCGACAA
KCND2	F: CATTGGGTGGATGCCTGTT R: CCGTGCGGTAGAAGTTGA
KCND3	F: TCCAGCGGACAAGAACA R: GGGTCACGGTCAAAGAAGTA
KCNE1	F: CCAATTCCACGACTGTTCTG R: AGGGTGAAGAAGCCGAAGA
CACNA1C	F: GTTTCGTCATTGTCACCTTCC R: TTGTACTGGTGCTGGTTCTTG
KCNH2	F: GTGCTGCCTGAGTATAAGCTG R: CCGAGTACGGTGTGAAGACT
GAPDH	F: CATCACTGCCACCCAGAAGACTG R: ATGCCAGTGAGCTTCCCGTTCAG

F, forward; R, reverse.

### 2.9 Isolated ventricular myocyte preparation

Mice were heparinized and anesthetized first; the hearts were then quickly excised and placed into precooled Ca^2+^-free Tyrode’s solution. After cannulation of the aorta, the heart was mounted on a Langendorff apparatus and perfused with Ca^2+^-free Tyrode’s solution [NaCl 137, KCl 5.4, MgCl_2_ 1.0, NaH_2_PO_4_ 0.33, D-glucose 10, and HEPES 10, (mM); pH 7.4] at 37°C for approximately 2 min. Then, the hearts were perfused with the same solution containing collagenase (Type II, Worthington, 450 U/mL), BSA (BBI, 1 mg/mL), protease (Type XIV, Sigma, 0.21 U/mL), and Ca^2+^ (50 μM) for 8–10 min. All solutions were equilibrated with 5% CO_2_/95% O_2_ and maintained at 37°C. When the tissues were well digested, the atria were removed, and ventricles were cut into small pieces and gently triturated to release single myocytes in B buffer (Ca^2+^-free Tyrode’s solution with 5 mg/mL BSA and 50 μM Ca^2+^). After filtering and centrifugation at 100 g, cells were stored in KB solution [L-glutamic acid 120, KOH 120, KCl 10, KH_2_PO_4_ 10, MgSO_4_ 1.8, EGTA 0.5, D-glucose 20, taurine 10, and HEPES 10, (mM); bovine serum albumin 0.2%; pH 7.2] at 4°C until Ca^2+^ (1.8 mM)-tolerant and ready for use. The obtained ventricular myocytes can be used simultaneously for patch clamp and intracellular calcium measurements.

### 2.10 Electrophysiological recording using the patch-clamp technique

Action potentials (APs) were recorded using the whole-cell patch-clamp technique under the current clamp mode (PatchMaster, EPC-10 amplifier, HEKA, Harvard Bioscience Inc, Reutlingen, Germany). The bath solution contained the following: NaCl 138, KCl 4, MgCl_2_ 1.0, NaH_2_PO_4_ 0.33, D-glucose 10, HEPES 10, and CaCl_2_ 2 (pH adjusted to 7.4 with NaOH) (mM). The pipette was filled with a solution of the following composition (mM): K-aspartate 120, KCl 20, MgCl2 1, HEPES 5, EGTA 5, and Mg-ATP 2 (pH adjusted to 7.2 with KOH). Action potentials were evoked by current pulses of 800 pA (4 ms in duration) at a frequency of 1 Hz. The time course of the action potentials was analyzed using pClamp software (Clampfit 11.3, Axon, Molecular Devices, Sunnyvale, California, United States). The action potential configuration was analyzed by measuring APD from the peak of the action potential to 10%, 30%, 50%, 70%, and 90% of repolarization.

Transient outward potassium current (I_to_) was recorded using the whole-cell patch-clamp configuration. Cells were superfused with a bath solution of the following composition (in mM): NaCl 146, KCl 4, MgCl_2_ 2.5, HEPES 5, dextrose 5.5, and CaCl_2_ 2 (pH adjusted to 7.3 with NaOH). The pipette was filled with a solution of the following composition (mM): KCl 135, EGTA 10, HEPES 10, dextrose 5.5, and Mg-ATP 3 (pH adjusted to 7.2 with KOH). Then, 20 μM nifedipine and 20 μM TTX were added to the bath solution to block the activity of I_Ca_ and I_Na_, respectively. After membrane rupture, the cell capacitance was measured, and the series resistance (Rs) was compensated electronically by 70%. I_to_ was evoked by 500-ms depolarizing pulses to potentials between −60 and +60 mV (10 mV steps) from a holding potential of −70 mV. The peak current density is calculated as I_to_. The amplitude and kinetics of I_to_ were analyzed using Clampfit 11.3 software. The L-type calcium current (I_Ca-L_) was recorded by using the whole-cell patch-clamp configuration. Ventricular myocytes were superfused with a bath solution of the following composition (in mM): NaCl 140, CaCl_2_ 1.8, CsCl 10, MgCl_2_ 1.0, glucose 10, and HEPES 10 (pH was adjusted to 7.4 with NaOH). The pipette was filled with a solution of the following composition (mM): CsCl 130, TEACl 20, HEPES 5.0, Na-GTP 0.1, EGTA 5.0, and Mg-ATP 5.0 (pH was adjusted to 7.2 with CsOH) (Zhang et al., 2024). The statistical analysis was carried out using GraphPad Prism 8.0.2. For AP, I_to_, and I_Ca-L_, patch electrode resistance was 3–5 MΩ. The recordings were conducted at room temperature (24 °C–26 °C).

### 2.11 Western blot

Total protein was extracted from ventricular tissues, with the entire procedure conducted on ice. The collected tissues were lysed with RIPA lysis buffer for 20 min. Lysates were centrifuged at 12,000 rpm at 4 °C for 15 min. The supernatant was stored at −80 °C after determining its protein concentration using Bradford assay. A measure of 20 μg protein for each lane was separated using 10% SDS-PAGE and transferred to a PVDF membrane (Millipore, United States). The membrane was incubated in TBST containing 5% non-fat milk for 2 h at room temperature to block nonspecific binding and was incubated with the primary antibody (Kv 4.2, 1:1,000, ab192762, Abcam, United States) overnight at 4 °C. The membrane was incubated with the horseradish peroxidase (HRP)-conjugated goat anti-rabbit or mouse IgG (1:3,000, BBI, China). The secondary antibody was incubated for 1 h at room temperature. The membrane was incubated in chemiluminescent HRP substrate (Millipore, United States) at room temperature for 30 s and then imaged using the Universal Hood II System (Bio-Rad, United States) (Eslami and Lujan, 2010).

### 2.12 Intracellular Ca^2+^ concentration quantification

[Ca^2+^]_i_ was quantified using Fluo-4 AM (Molecular Probes, Thermo Fisher Scientific, United States). In brief, ventricular myocytes were placed in tubes containing Fluo-4 AM (5 μmol/L reconstituted in DMSO) along with 0.1% Pluronic F-127 (P2443, Thermo Fisher Scientific, United States) for 30 min in the dark and then washed for 20 min in Tyrode’s solution. The ventricular myocytes were then placed on coverslips and perfused with Tyrode’s solution (1.8 mM Ca^2+^) at room temperature for 10 min. Images were obtained by the line scan mode using a confocal microscope (LSM980, Carl Zeiss, German). The ventricular myocytes were field-stimulated at 0.5 Hz. A wavelength of 488 nm was applied to excite the samples and collect fluorescence emission spectra at 505 nm (40 nm band-pass filter) for single-color calcium imaging. To standardize the fluorescence intensity of intracellular Ca^2+^ indicators, the change in [Ca^2+^]_i_ (∆[Ca^2+^]_i_) was expressed as ΔF/F0 = (F − F0)/F0. Here, F0 is a measure of the baseline fluorescence at the resting state, F is the fluorescence intensity at any given time, and ∆F is the moment-by-moment deviation from that baseline (Li et al., 2023a).

### 2.13 Statistical analysis

All data are expressed as mean ± standard deviation. Differences between group means were examined using two-tailed, unpaired Student’s t-test or two-way analysis of variance (ANOVA) with Sidak’s test for multiple comparisons. *P* < 0.05 was considered statistically significant.

## 3 Results

### 3.1 CIT induces QT interval prolongation and ECG abnormalities without alternation of cardiac hemodynamics or structure

**FIGURE 1:**
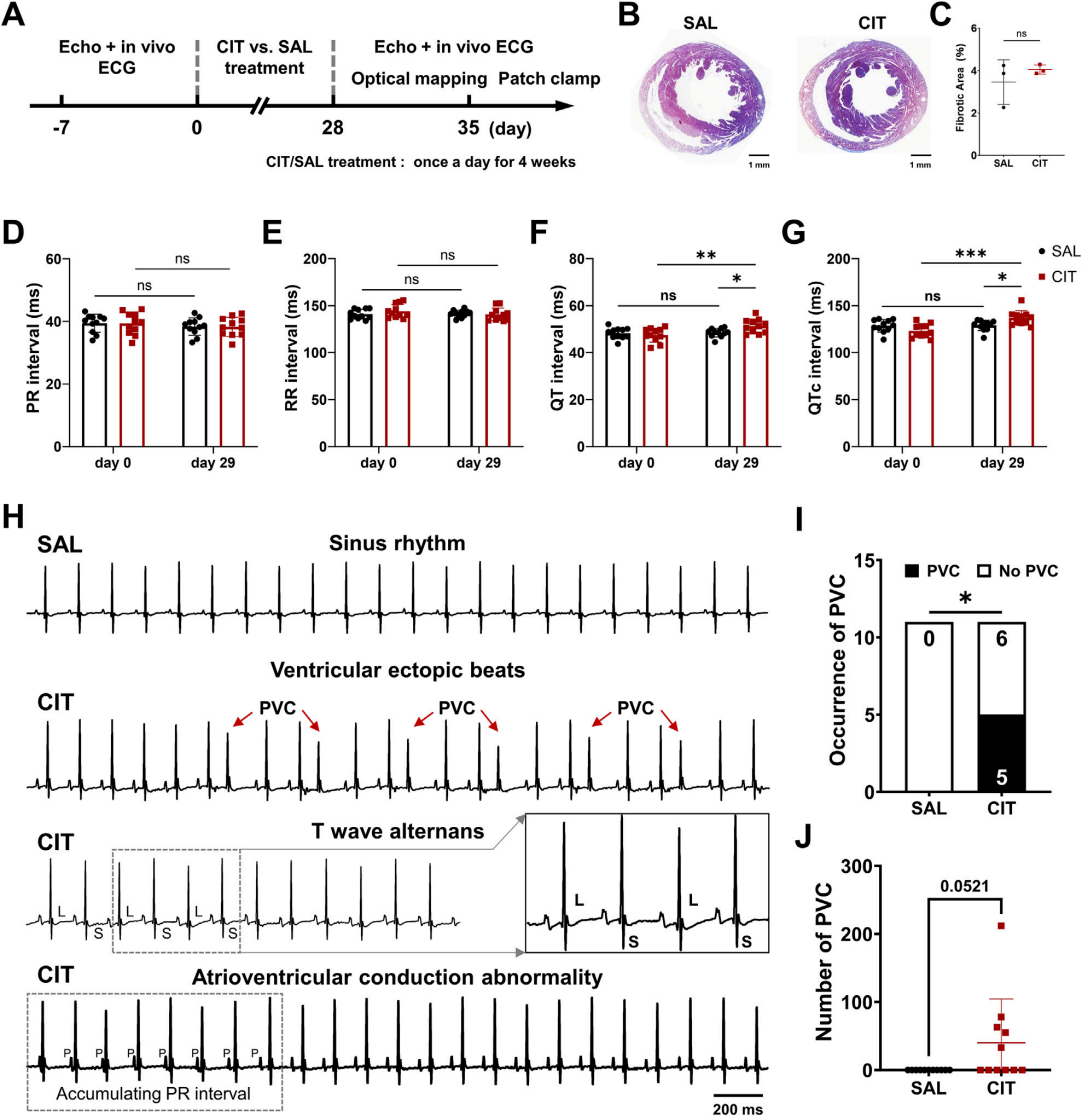
*In vivo* ECG monitoring and evaluation of heart function following 4 weeks of treatment with citalopram and saline. **(A)** Timeline of the study treatment with citalopram across multiple experiments. **(B)** Representative transverse cardiac slice stained with Masson’s trichrome. **(C)** Statistical percentage of the fibrosis area. **(D)** Statistical results of the PR interval; **(E)** RR interval; **(F)** QT interval; **(G)** QTc interval. **(H)** Representative ECG traces before (day 0) and after 4 weeks of CIT treatment. **(I)** Occurrence of spontaneous PVCs. **(J)** Number counts of PVCs. (^*^ *P* < 0.05 and ^**^ *P* < 0.01, SAL vs. CIT mice, n = 11 for each group). ^*^ *P* < 0.05. Student’s t-test, chi-square test with Fisher’s exact test, and two-way analysis of variance (ANOVA) with Sidak’s test for multiple comparisons. CIT, citalopram; SAL, saline; ECG, electrocardiogram; QTc, corrected QT; PVC, premature ventricular contraction; L, large; S, small; P, P wave.

As shown in Figure 1A, Echo and Masson staining showed that CIT did not significantly induce hemodynamic or structural remodeling of the hearts (Figures 1B,C; Table 2). Surface ECG recordings *in vivo* revealed that mice undergoing CIT treatment presented significant QT interval prolongation (day 29 SAL: 48.49 ± 1.94 ms vs. day 29 CIT: 51.31 ± 2.92 ms, *P* = 0.0138; day 0 CIT: 47.45 ± 2.98 ms C vs. day 29 CIT: 51.31 ± 2.92 ms, *P* = 0.0011) (Figures 1F,G) compared with those treated with SAL, while the PR interval and RR interval were not significantly altered (Figures 1D,E). In addition to the effects on QT interval prolongation, CIT-treated mice also exhibited multiple abnormalities in ECG recordings and higher incidence of spontaneous premature ventricular beats (PVCs), as shown in Figures 1H,I. All mice displayed a normal sinus rhythm at the baseline; however, following CIT intervention, electrical abnormalities such as PVCs, T wave alternans (TWA), and atrioventricular conduction abnormalities were recorded, which indicated the proarrhythmic effect of CIT. Then, the occurrence and number count of PVCs were quantified in both SAL and CIT groups during 15 min. Five of eleven mice showed spontaneous PVCs in the CIT group (day 0 CIT: 0/11 vs. day 29: 5/11, *P* = 0.0351), with an average of approximately 50 PVCs for each mouse (an average of 100 PVCs for the five mice with electrical abnormalities), while no PVCs were observed in the SAL group (Figure 1J).

**TABLE 2:** Echocardiogram and growth characteristic testing after 4 weeks of CIT and SAL treatment.

Parameters	SAL	CIT
EF (%)	55.41 ± 4.75	57.10 ± 3.22
FS (%)	29.11 ± 2.43	29.59 ± 2.17
IVSd (mm)	0.82 ± 0.05	0.85 ± 0.04
IVSs (mm)	1.22 ± 0.06	1.27 ± 0.08
LVIDd (mm)	3.80 ± 0.25	3.94 ± 0.17
LVIDs (mm)	2.85 ± 0.20	2.87 ± 0.13
LVPWd (mm)	0.70 ± 0.03	0.67 ± 0.04
LVPWs (mm)	0.96 ± 0.07	0.96 ± 0.08
HW/TL (mg/mm)	6.38 ± 0.41	6.25 ± 0.33
HW/BW (mg/g)	5.43 ± 0.42	5.56 ± 0.56

EF, ejection fraction; FS, fractional shortening; IVSd, interventricular septal dimension at end-diastole; IVSs, interventricular septal dimension at end-systole; LVIDd, left ventricular internal dimension at end-diastole; LVIDs, left ventricular internal dimension at end-systole; LVPWd, left ventricular posterior wall thickness at end-diastole; LVPWs, left ventricular posterior wall thickness at end-systole; HW, heart weight; TL, tibial length; BW, body weight. Data are illustrated as mean ± SD (n = 5 per group for echocardiogram and n = 8 per group for HW/BW).

**FIGURE 2:**
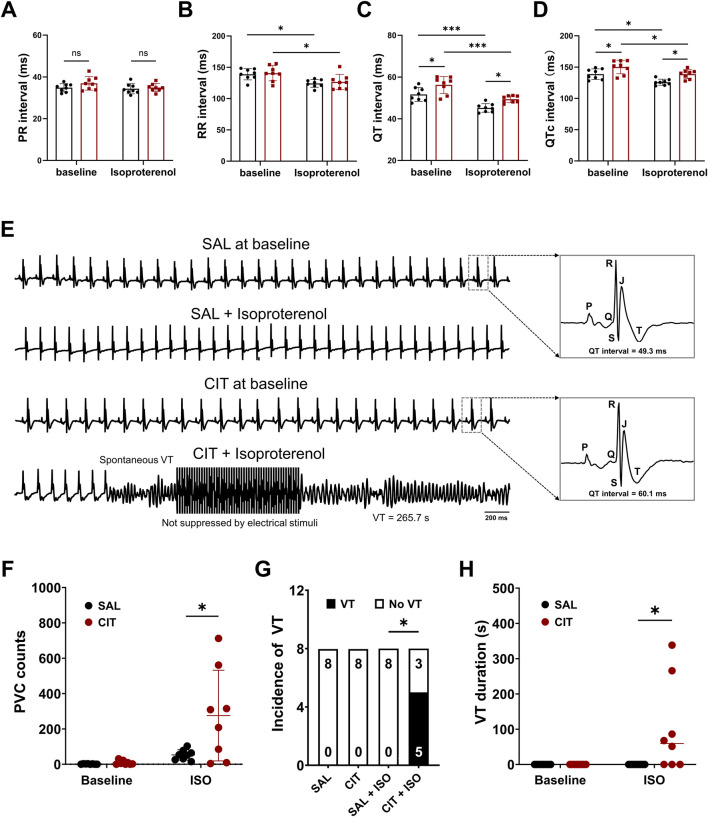
*Ex vivo* ECG results after 4 weeks of citalopram and saline treatment. **(A)** Statistical results of the PR interval; **(B)** RR interval; **(C)** QT interval; **(D)** QTc interval. **(E)** Representative ECG traces before and after ISO challenge. **(F)** Number counts of spontaneous PVCs. **(G)** Incidence of VT and **(H)** total VT duration. ^*^ *P* < 0.05. Student’s t-test and chi-square test with Fisher’s exact test. VT, ventricular tachycardia.

Consistent with the *in vivo* surface ECG results, both the QT interval and the QTc interval were significantly prolonged in isolated hearts from mice treated with CIT *in vitro*, while the PR and RR intervals were not affected (Figures 2A,B). Under ISO challenge, the PR interval remained unchanged, while the RR interval was significantly reduced (SAL: 138.9 ± 8.98 ms vs. SAL + ISO: 124.2 ± 6.23 ms, *P* = 0.0366; CIT: 140.5 ± 12.15 ms vs. CIT + ISO: 126.5 ± 12.17 ms, *P* = 0.0490; n = 8 for each group). Meanwhile, the QT interval and QTc interval were also reduced by ISO treatment (QT interval: SAL: 51.66 ± 3.30 ms vs. SAL + ISO: 45.19 ± 2.07 ms, *P* = 0.0007; CIT: 56.21 ± 4.04 ms vs. CIT + ISO: 49.40 ± 1.63 ms, *P* = 0.0004; n = 8 for each group. QTc interval: SAL: 138.7 ± 8.35 ms vs. SAL + ISO: 125.8 ± 4.75 ms, *P* = 0.0176; CIT: 150.2 ± 10.93 ms vs. CIT + ISO: 138.8 ± 7.15 ms, *P* = 0.0417; n = 8 for each group). Moreover, hearts isolated from mice treated with CIT showed an increased incidence of ventricular arrhythmias, manifested as PVCs and VT (Figures 2E–G, SAL 0/8 vs. CIT 5/8, *P* = 0.0256). Representative ECG recordings are shown in Figure 2E; sustained polymorphic ventricular tachycardias (PVTs) were induced in CIT-treated hearts with significantly prolonged QT intervals (QT interval = 60.1 ms), while the control hearts presented normal QT intervals (QT interval = 49.3 ms) and maintained sinus rhythm before and after ISO intervention. Then, these abnormal cardiac events were quantified as shown in Figures 2F,H; CIT-treated hearts presented higher PVC counts (SAL 52.75 ± 29.05 vs. CIT 275.4 ± 256.4, *P* = 0.0286; n = 8 for each group) and longer VT duration (SAL 0 vs. CIT 101.1 ± 129.7, *P* = 0.0447; n = 8 for each group). These data suggested that CIT treatment would induce prolonged QT intervals and higher vulnerability of ventricular arrhythmias in the absence of cardiac structural remodeling.

### 3.2 CIT treatment prolongs APD and CaTD accompanied by altered voltage–calcium coupling

**FIGURE 3:**
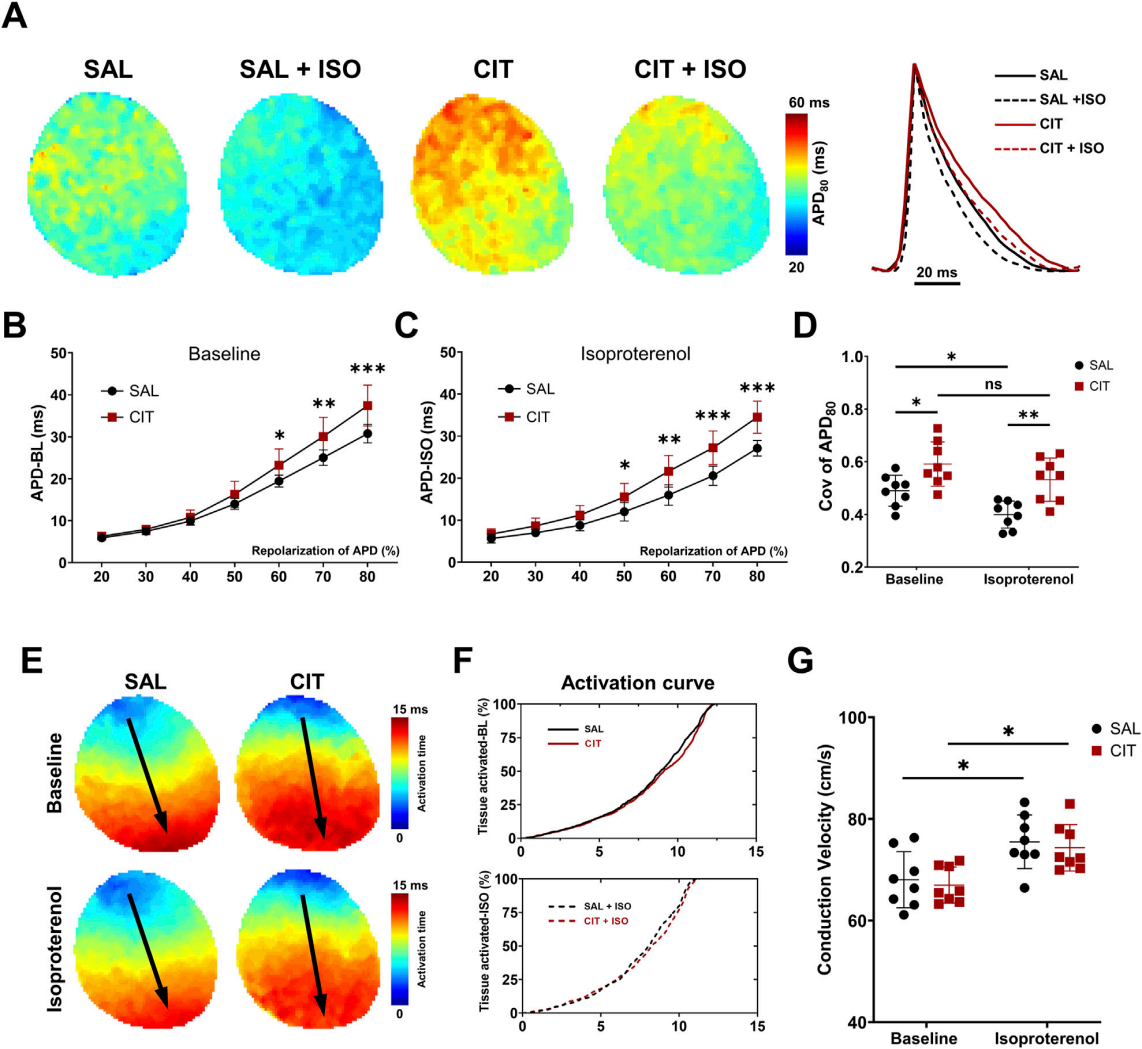
APD properties and conduction analysis in SAL- and CIT-treated hearts. **(A)** Representative APD_80_ maps and corresponding AP traces before and after ISO intervention (1 μM) in CIT-treated hearts vs. SAL-treated hearts. **(B)** Statistical results of APD (repolarization percentage of 20%–80%) at baseline and **(C)** after ISO challenge at 10 Hz. **(D)** Heterogeneity of APD_80_ in CIT-treated hearts vs. SAL hearts. **(E)** Representative activation maps in CIT hearts vs. SAL hearts before and after ISO challenge. **(F)** Activation curves of activated tissue percentage. **(G)** Quantified conduction velocity in CIT-treated hearts vs. SAL-treated hearts (CV baseline: 68.03 ± 5.511 cm/s vs. CIT: 66.96 ± 3.544 ms, P = 0.998; isoproterenol: 75.48 ± 5.272 ms vs. CIT: 74.31 ± 4.569 ms, P = 0.997). ^*^ *P* < 0.05, ^**^ *P* < 0.01, and ^***^ *P* < 0.005. Two-way analysis of variance (ANOVA) with Sidak’s test for multiple comparisons. APD, action potential duration; AP, action potential; ISO, isoproterenol; CV, conduction velocity.

**FIGURE 4:**
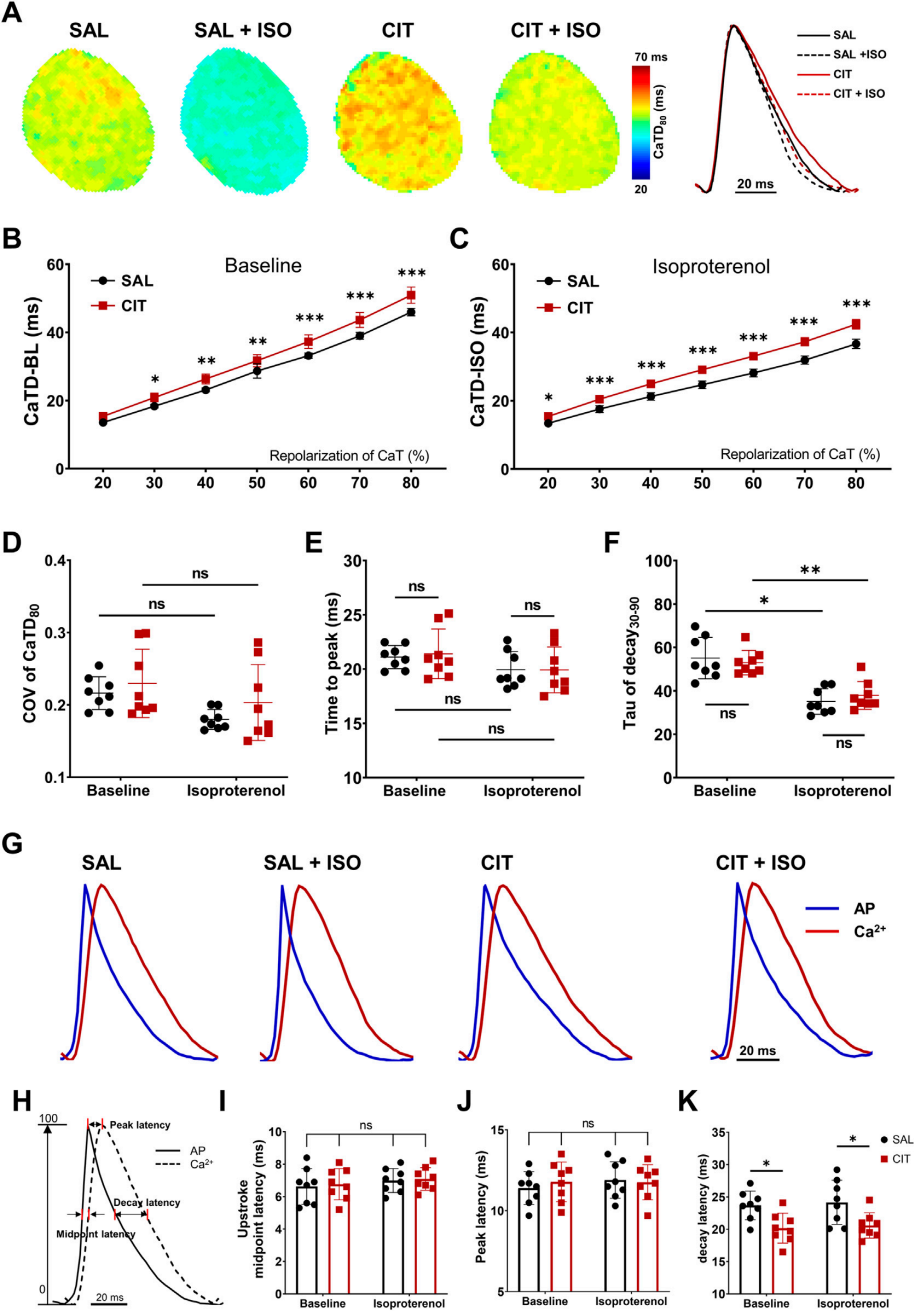
Prolonged CaTD and impaired Vm–Ca^2+^ coupling in CIT-treated hearts. **(A)** Representative CaTD_80_ maps and corresponding AP traces before and after ISO intervention (1 μM) in CIT-treated hearts vs. SAL-treated hearts. **(B)** Statistical results of CaTD (repolarization percentage of 20%–80%) at baseline and **(C)** after ISO challenge. **(D)** Heterogeneity of CaTD_80_ in CIT-treated hearts vs. SAL hearts. **(E)** Statistical results of time to peak in CIT-treated hearts vs. SAL hearts. **(F)** Tau of decay analysis in CIT-treated hearts vs. SAL-treated hearts. **(G)** Representative AP and CaT coupling traces before and after ISO challenge. **(H)** Schematic of the voltage–calcium latency algorithm. **(I)** Statistical results of upstroke midpoint latency. **(J)** Statistical results of peak latency. **(K)** Statistical results of voltage–calcium decay latency. n = 8 for each group. ^*^ *P* < 0.05, ^**^ *P* < 0.01, and ^***^ *P* < 0.005. Two-way analysis of variance (ANOVA) with Sidak’s test for multiple comparisons. CaTD, calcium transient duration.

**FIGURE 5:**
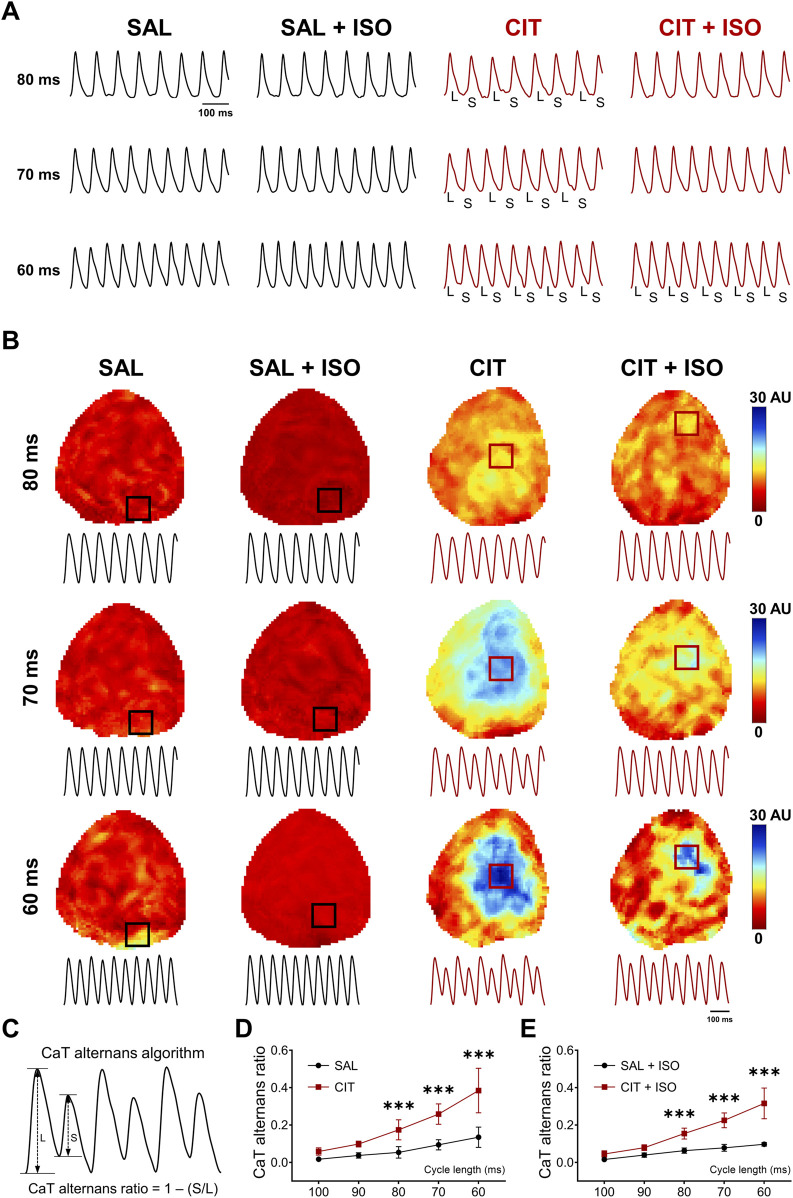
Aggravated cardiac alternans in CIT-treated hearts. **(A)** Typical optical AP traces of APD alternans. **(B)** Representative CaT alternans maps and corresponding traces before and after ISO challenge in SAL- and CIT-treated hearts. **(C)** Sketch diagram of CaT alternans ratio. **(D)** Quantification of CaT alternans in SAL- and CIT-treated hearts at baseline and **(E)** after ISO challenge. ^*^ *P* < 0.05, ^**^ *P* < 0.01, and ^***^ *P* < 0.005. Two-way analysis of variance (ANOVA) with Sidak’s test for multiple comparisons.

**FIGURE 6:**
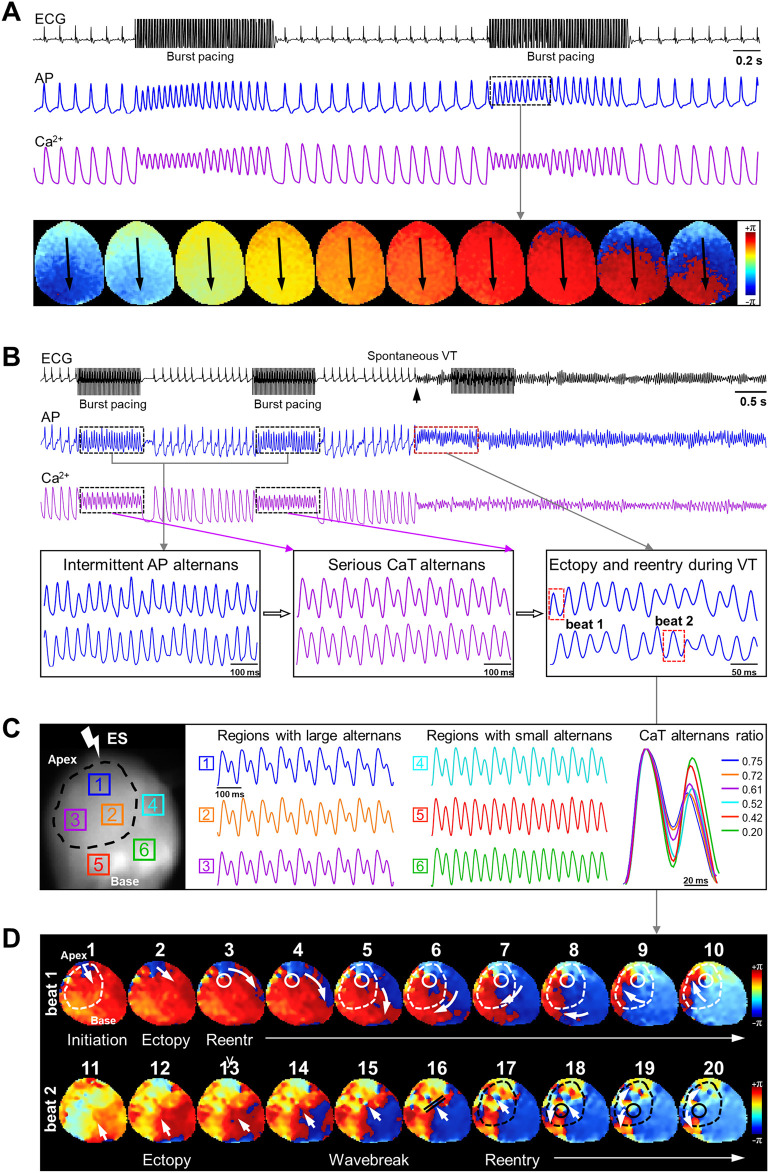
Integrated analysis of cardiac alternans and phase maps of reentrant arrhythmias in SAL- and CIT-treated hearts. **(A)** Typical phase maps of a SAL-treated heart under burst pacing. **(B)** ECG recordings and corresponding AP and CaT traces under 50 Hz burst pacing; APD alternans were induced accompanied by CaT alternans. **(C)** Serious CaT alternans were restricted in the black circle region. **(D)** PS trajectory was overlapped with the region with most serious alternan. PS, phase singularity.

Proarrhythmic electrophysiological mechanisms of CIT were explored using cardiac optical mapping (Figures 3–6). Representative action potential duration at 80% repolarization (APD_80_) maps and corresponding optical traces before and after ISO application are illustrated in Figure 3A. The APD in hearts treated with CIT was significantly prolonged before and after ISO challenge compared with the control hearts (APD_80_ baseline: 30.76 ± 2.78 ms vs. CIT: 37.44 ± 4.88 ms, *P* < 0.0001; APD_80_ isoproterenol: 27.12 ± 1.85 ms vs. CIT: 34.51 ± 3.80 ms, *P* < 0.0001; n = 8 for each group) (Figures 3B,C). APD dispersion was increased in CIT-treated hearts. ISO reduced APD dispersion in SAL-treated hearts, but not in CIT-treated hearts (baseline: 0.490 ± 0.064 vs. CIT: 0.591 ± 0.084, *P* = 0.037; isoproterenol: 0.399 ± 0.051 vs. CIT: 0.532 ± 0.083, *P* = 0.0042; n = 8 for each group) (Figure 3D). Since studies have revealed that Nav 1.5 was influenced in CIT-related cardiac toxicity (Nakatani and Amano, 2021), the conduction of action potential was then assessed. ISO was effective in increasing the conduction velocity (CV) in both groups, while no difference between SAL- and CIT-treated hearts was found (Figures 3E–G).

Meanwhile, intracellular calcium-transient signals simultaneously recorded with membrane voltage (V_m_) were investigated. The calcium transient duration at 80% repolarization (CaTD_80_) maps and corresponding traces under programmed 10 Hz S1S1 pacing are shown in Figure 4A. The results suggested that CIT treatment significantly prolonged CaTD_80_ (Figures 4B,C) without altering CaTD_80_ heterogeneity (Figure 4D) when compared with SAL treatment (CaTD_80_ baseline: 45.94 ± 1.09 ms vs. CIT: 50.92 ± 2.40 ms, *P* < 0.0001; ISO: 36.67 ± 1.35 ms vs. CIT + ISO: 42.42 ± 1.39 ms, *P* < 0.0001; n = 8 for each group). ISO decreased the time constant (tau) of decay in both groups, but no difference was observed in the tau of decay and time to peak (TTP) between SAL- and CIT-treated hearts (Figures 4E,F). We further investigated the physiological delay between AP and CaT (voltage–calcium latency) at three time points: the upstroke midpoint latency, the peak latency, and the decay latency, as shown in Figure 4H. Representative AP and CaT coupling traces at baseline and under ISO challenge are shown in Figure 4G; the peak and upstroke midpoint latencies (Figures 4I,J) were not altered with CIT treatment. However, the decay latency of CIT-treated hearts was significantly shortened compared with SAL hearts (Figure 4K), suggesting an alteration or turbulence of the relationship between AP and CaT (baseline: 23.69 ± 2.805 ms vs. CIT: 20.16 ± 2.310 ms, *P* = 0.045; ISO: 24.16 ± 3.411 ms vs. CIT + ISO: 20.59 ± 1.891 ms, *P* = 0.041; n = 8 for each group). The different degree of APD and CaTD prolongation resulted in impaired Vm–Ca coupling that may interrupt the physiological orchestrated relationship of membrane currents and intracellular calcium handling.

### 3.3 CIT aggravates AP and CaT alternans in isolated hearts

Since AP and CaT alternans are widely believed to be predictors of lethal arrhythmias (Kanaporis and Blatter, 2023), programmed S1S1 pacing from a cycle length of 100 to 50 ms was conducted to induce cardiac alternans, according to previous studies (Fukaya et al., 2019; Liao et al., 2021). Figure 5A presents representative APD alternans traces at 80–60 ms at baseline and after ISO challenge. CIT-treated hearts showed frequency-dependent APD alternans. Meanwhile, accompanied by APD alternans, CIT-treated hearts also showed frequency dependence and suffered larger and more severe CaT alternans in a broader region among all the pacing frequencies (Figure 5B). The CaT alternans algorithm is illustrated in Figure 5C. CaT_Large_ (L) and CaT_Small_ (S) are the amplitudes of the large and small CaTs of a pair of alternating CaTs, respectively. The CaT alternans ratio = 1 − S/L. By this definition, the CaT alternans ratio values fall between 0 and 1, where the CaT alternans ratio = 0 indicates no Ca^2+^ alternans and CaT alternans ratio = 1 indicates a situation where SR Ca^2+^ release is completely abolished on every other beat. CaTs were considered alternating when the beat-to-beat difference in CaT amplitude exceeded 10% (CaT alternans ratio > 0.1) (Kanaporis and Blatter, 2023). Quantification of CaT alternans showed that CIT-treated hearts possessed a higher CaT alternans ratio than the SAL-treated hearts at 80–60 ms (Figures 5D,E). Another CaT alternans algorithm quantified the duration of CaT(n)/CaT(n + 1), and the ratio reflects the degree of calcium alternation well as the ratio of 1 − S/L (Supplementary Figure S2). APD alternans accompanied by CaT alternans were deteriorated in CIT-treated hearts, which promote CIT-related arrhythmias.

### 3.4 CIT-induced cardiac alternans predisposes to risk of ventricular tachyarrhythmias

Since CIT-treated hearts exhibited significant frequency-dependent AP and CaT alternans, we investigated the relationship between alternans and arrhythmias in CIT-treated hearts, especially CaT alternans. Representative ECG and corresponding AP and CaT traces under burst pacing in a SAL-treated heart are shown in Figure 6A; the normal sinus rhythm was maintained at baseline and after burst pacing without AP or CaT alternans. Phase maps indicated that AP conduction from the pacing region to the bottom was even and synchronous. However, as shown in Figure 6B, the CIT-treated heart exhibited spontaneous and sustained VT after two sequences of 50 Hz burst pacing. In further investigation of corresponding AP and CaT signals, severe CaT alternans with intermittent AP alternans were observed during fast pacing. Based on previous research studies on CaT alternans and arrhythmias, spatial discordant alternans (SDA) is believed to be firmly associated with arrhythmias (Aras et al., 2022; Chien et al., 2023); therefore, the spatial heterogeneity of CaT alternans with subsequent reentry activities were explored. Figure 6C shows six regions exhibiting large (regions 1, 2, and 3) and small (regions 4, 5, and 6) CaT alternans. By the combination analysis of phase maps and spatial properties of CaT alternans, the phase singularities (PSs) were found to be rotating and meandering in regions 1, 2, and 3 (Figure 6D), even in the absence of SDA. Regions with the most severe alternans overlapped with the PS trajectory in the reentrant circuits. These findings, to some extent, also indicated that the “spatial discordance” of CaT alternans was a potential electrophysiological substrate of CIT-related tachyarrhythmias. The dynamic process of VT initiation and maintenance of VT is shown in Figure 6D. In beat 1 of the upper panel, the ectopy was generated from the apex of the heart first, and the ectopic action potentials spread to the bottom rapidly. However, with impaired function caused by serious CaT alternans, the electrical pulses were terminated at the circle region border and shifted to the right side to escape from these regions with higher refractoriness, thus causing a wave-break and the formation of the phase singularity (Handa et al., 2020), which led to reentrant activities. The reentry circuits meandered around the PS and did not stop until the PS shifted and generated another ectopy in beat 11 shown in Figure 6D. A phase map (Figure 6D beat 11–20) showed the identical process of the maintenance of reentry activity; the trajectory of the PS existed in the dotted region for much of the duration in the subsequent VT episodes. Regional differences in CaT alternans initiated ectopic beats and led to reentry activities to maintain the sustained VT.

### 3.5 CIT induces the downregulation of expression of multiple potassium channels

**FIGURE 7:**
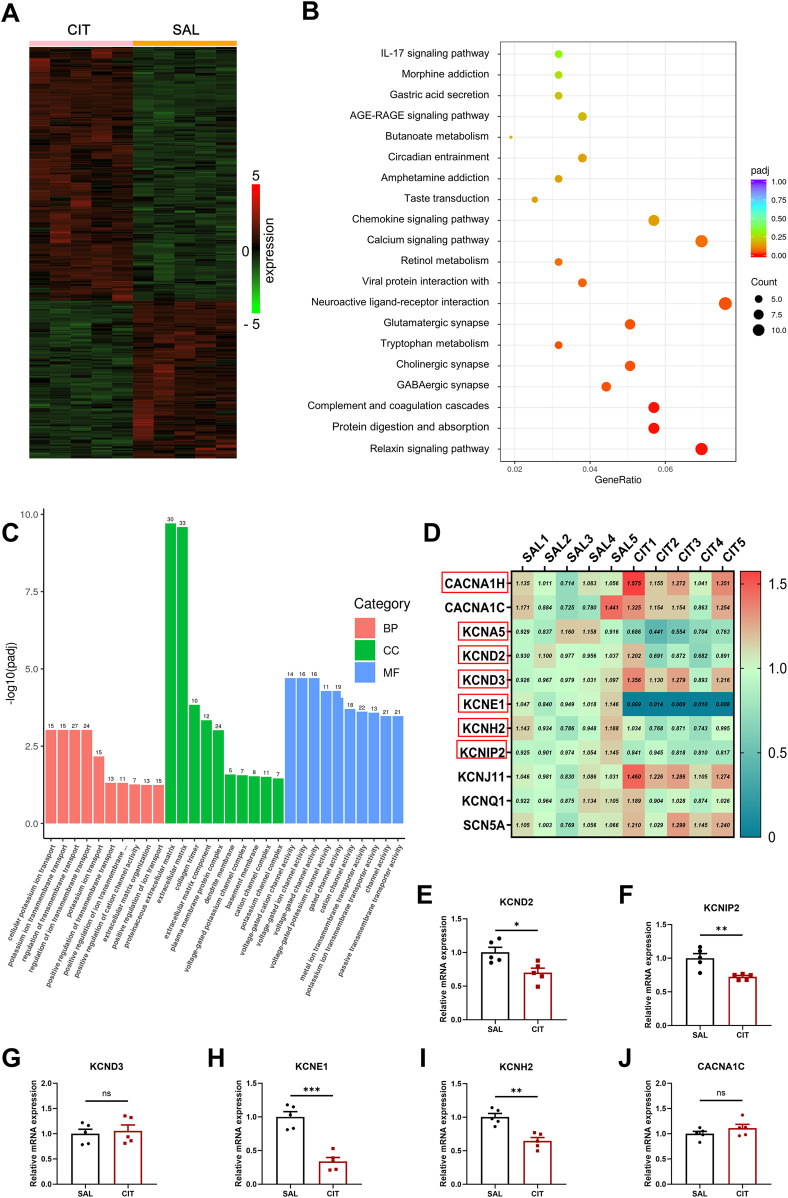
Transcriptomic analysis and RT-qPCR results in CIT-treated ventricular tissue. **(A)** Heat map of gene clusters in SAL-/CIT-treated hearts. **(B)** KEGG pathway enrichment analysis of differentially expressed genes. **(C)** GO enrichment analysis of differentially expressed genes. **(D)** Analysis on the expression of main ion channels related to multiple potassium channels. **(E–J)** RT-qPCR statistical results in SAL-/CIT-treated hearts. ^*^ *P* < 0.05, ^**^ *P* < 0.01, and ^***^ *P* < 0.005. Student’s t-test and Fisher’s exact test. KEGG, Kyoto Encyclopedia of Genes and Genomes; GO, Gene Ontology; RT-qPCR, real-time quantitative polymerase chain reaction.

To further investigate the underlying mechanisms of CIT-induced arrhythmias, the transcriptomics array was carried out using cardiac ventricular tissue from mice undergoing different treatments (Figure 7). A heat map of gene clusters in each group is displayed in Figure 7A. The results revealed that CIT treatment induced alterations in gene clusters. KEGG and GO enrichment analyses of differentially expressed genes (DEGs; *P* < 0.05 and Log_2_ fold of change > 1) demonstrated that DEGs were enriched in pathways including the calcium signaling pathway, cellular potassium ion transport, potassium channel complex, and regulation of ion transmembrane transport, which may contribute to the effects of CIT on cardiac electrophysiology and arrhythmias (Figures 7B,C; Supplementary Table S1).

Further analysis on the expression of main ion channels revealed that expressions of multiple potassium channels were downregulated (Figure 7D). Subsequently, the expressions of genes involved in K^+^ and Ca^2+^ channels were confirmed using RT-qPCR (Figure 7E). These data revealed that KCND2, KCNH2, KCNIP2, and KCNE1 were remarkably reduced following treatment with CIT (*P* < 0.05), while the expressions of CACNA1C and KCND3 were not significantly altered.

### 3.6 Downregulation of I_to_ and I_Ca-L_ contributes to CIT-induced APD prolongation

**FIGURE 8:**
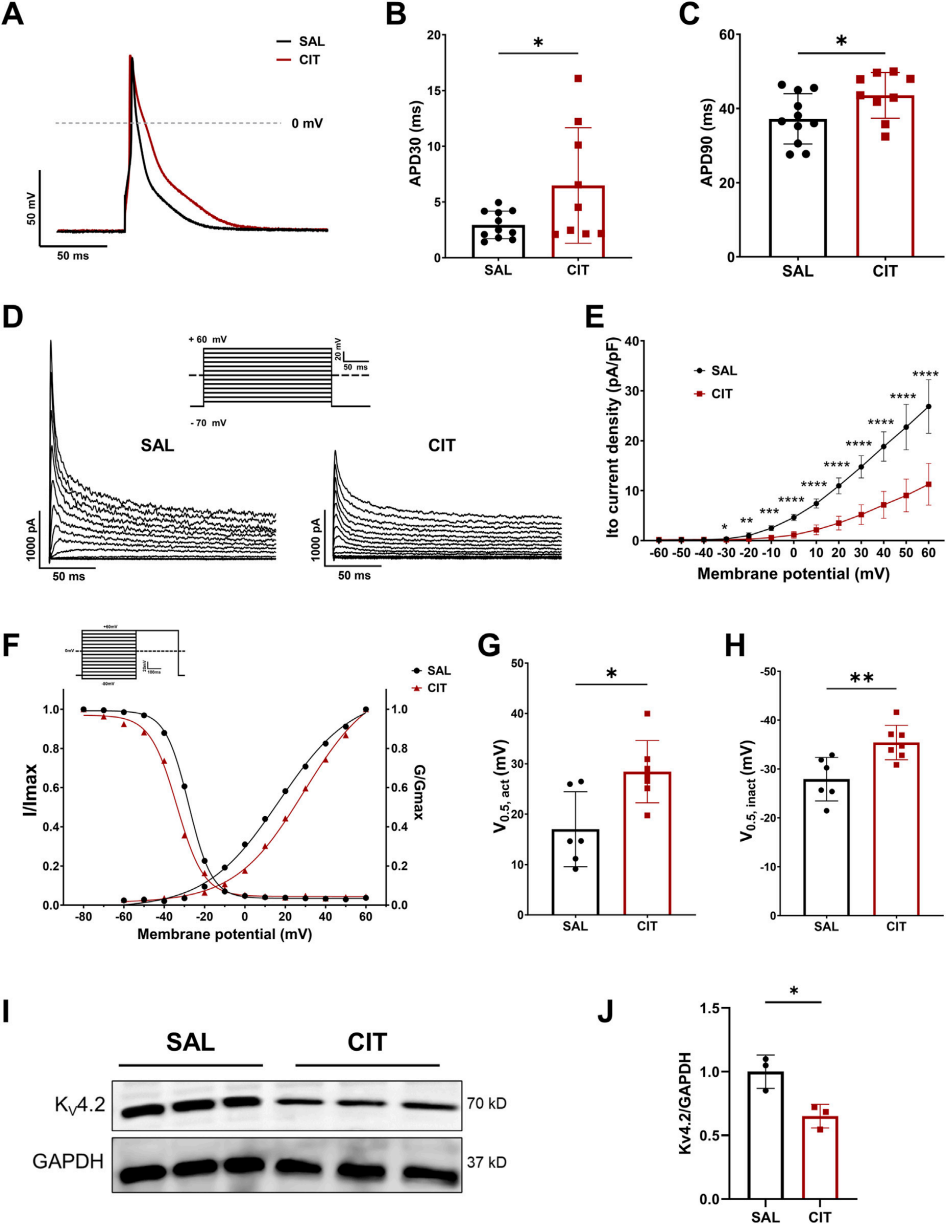
Effects of CIT treatment on action potential (AP) and transient outward K+ current (*I*_to_) in mouse ventricular myocytes. **(A)** Representative traces of action potentials at 1 Hz stimulation of CIT and SAL groups. **(B)** Action potential durations at 30% (APD_30_) repolarization. (SAL: n = 11 myocytes and 4 mice; CIT: n = 9 myocytes and 3 mice). **(C)** Action potential durations at 90% (APD_90_) repolarization. (SAL: n = 11 myocytes and 4 mice; CIT: n = 9 myocytes and 3 mice). **(D)** Representative current traces of *I*_to_ in CIT and SAL ventricular myocytes. **(E)** Means ± SE *I*_to_ density from CIT and SAL groups (SAL: n = 6 myocytes and 3 mice; CIT: n = 7 myocytes and 3 mice; ^*^ *P* < 0.05; ^**^ *P* < 0.01; ^***^ *P* < 0.001; ^****^ *P* < 0.0001). **(F)** Voltage-dependent activation and inactivation of *I*_to_ curves in CIT and SAL groups. Average half-maximal voltage of activation and **(G)** inactivation **(H)**. **(I)** Original image of Western blotting showing the band of Kv4.2. **(J)** Bar graphs of the Kv4.2 protein expression level, n = 3; ^*^ *P* < 0.05.

Action potentials were further measured using the patch-clamp technique at the single-cell level for deeper mechanism searching. Examples of APs recorded in isolated cells from CIT and SAL ventricles (at 1 Hz) are shown in Figure 8A. APD_30_ of CIT-treated cardiomyocytes was significantly prolonged compared with SAL-treated cardiomyocytes, (6.481 ± 5.183 ms vs. 2.941 ± 1.241 ms, *P* < 0.05, Figure 8B). APD_90_ of CIT-treated cardiomyocytes was also significantly prolonged compared with SAL-treated cardiomyocytes (*P* < 0.05, Figure 8C). In agreement with APD prolongation, the mean *I*_to_ density was significantly reduced in CIT-treated cardiomyocytes, and the *I*_to_ stimulation protocol and representative traces are shown in Figures 8D,E. Changes in the rates of activation and inactivation of *I*_to_ were quantified. CIT treatment caused a positive shift in the voltage-dependent activation and a negative shift in inactivation (Figure 8F). The half-maximal voltage of activation (V_0.5, act_) estimated by fitting the activation curve with a Boltzmann function was shifted from 28.42 ± 2.336 to 17.00 ± 3.040 mV in cells treated with CIT (*P* < 0.05, Figure 8G). The voltages at half-maximum inactivation (V_0.5, inact_) estimated by fitting the inactivation curve with a Boltzmann function were shifted from −35.43 ± 1.330 to −27.92 ± 1.814 mV in cells treated with CIT (*P* < 0.01, Figure 8H), illustrating that CIT treatment changed *I*_to_ gating kinetics and regulated the voltage dependence of *I*_to_ activation and inactivation, which led to a prolongation of APD in ventricular cardiomyocytes. To further clarify the protein expression after CIT treatment, we then conducted Western blot and found that the Kv4.2 expression level was significantly reduced (*P* < 0.05, Figures 8I,J), which was consistent with the decreased mRNA expression of KCND2.

**FIGURE 9:**
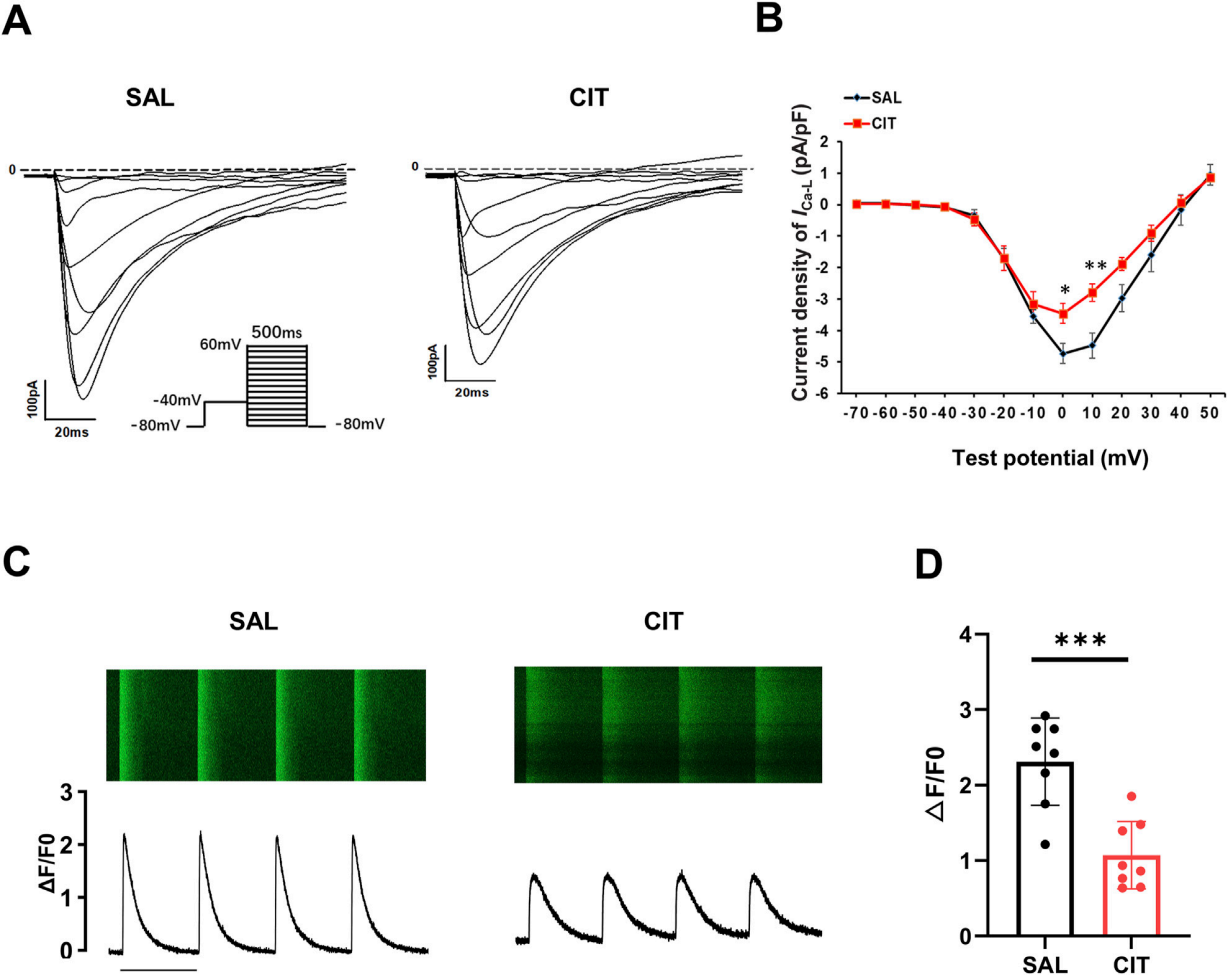
Decreased I_Ca-L_ current and intracellular calcium amplitude after CIT treatment. **(A)** Representative current traces of I_Ca-L_ in CIT- and SAL-treated ventricular myocytes. **(B)** Statistical results of I_Ca-L_ current density in CIT- and SAL-treated ventricular myocytes. (SAL: n = 8 myocytes and 2 mice; CIT: n = 12 myocytes and 3 mice; *P < 0.05; **P < 0.01). **(C)** Typical Ca^2+^ transient images and corresponding CaT traces from CIT- and SAL-treated ventricular myocytes, bar = 2 s. **(D)** Statistical results of relative intracellular calcium amplitude by line scan. (SAL: n = 8 myocytes and 2 mice; CIT: n = 8 myocytes and 3 mice; * * * *P* < 0.001).

The aforementioned data suggest that the abnormal Ca^2+^ handling is the effect of CIT on the prolongation of CaTD, which prompted us to further investigate whether CIT can directly affect I_Ca-L_ and intracellular calcium. The results showed that CIT reduced I_Ca-L_ current density (Figure 9A). In particular, CIT reduced the peak current density of I_Ca-L_ (at 0 mV) from −4.73 ± 0.32 pA/pF to −3.46 ± 0.31 pA/pF (*P* = 0.013) and (at 10 mV) from −4.48 ± 0.40 pA/pF to −2.80 ± 0.28 pA/pF (*P* = 0.002, Figure 9B). Next, Δ[Ca^2+^]_i_ was monitored using the Fluo-4 AM indicator. The results showed that ∆[Ca^2+^]i was significantly reduced in CIT-treated cardiomyocytes, with ΔF/F0 from 2.31 ± 0.20 to 1.1 ± 0.16 (Figures 9C,D).

## 4 Discussion

### 4.1 Prolonged APD and CaTD with impaired E–C coupling in CIT-treated hearts

Previous studies have found that citalopram and escitalopram cause prolongation of the QT interval on ECG and pathologic QTc prolongation in some patients (Akturk et al., 2023; Faraj et al., 2023; Chen et al., 2024). At the same time, CIT carries the risk of TdP (Krøll et al., 2022). The cardiac side effects of QT interval prolongation and higher risk of TdP by citalopram were reproduced and validated in our current work. Subsequent optical mapping studies suggested that APD was significantly prolonged in CIT-treated hearts, which is consistent with results of other studies on drug-induced long QT syndrome. Generally speaking, prolonged APD provides the electrical substrate for generating early after depolarization (EAD) or delayed after depolarization (DAD), which may deteriorate into life-threatening tachyarrhythmias (Bersell et al., 2023; Chien et al., 2023). These results, in some aspects, demonstrate the proarrhythmic potential of CIT treatment. Increased APD dispersion was observed in CIT-treated hearts, which may cause discordant repolarization and result in lethal cardiac events (Khwaounjoo et al., 2022; Yamazaki et al., 2022). CaTD was also found to be prolonged before and after ISO challenge. Despite the regional heterogeneity in APD between CIT-treated hearts and controls, we did not find spatial differences in CaTD, similar to a recent study (Wang et al., 2020). Accumulating evidence over the past few decades has revealed that normal cardiac function resulted from sophisticated interactions between ion channels on the membrane and intracellular calcium-handling proteins; disturbance of these interactions may lead to severe arrhythmias (Lei et al., 2018; Lei and Huang, 2020). As shown in Figure 4G, APD and CaTD of CIT-treated hearts were prolonged to different extents compared with SAL-treated hearts, which, in turn, resulted in shortened voltage–calcium decay latency. The shortened Vm–Ca^2+^ decay latency did not imply a more inseparable but aggravated coupling relationship because the physiological decay was not completed during the diastolic interval (Zhou et al., 2016). Therefore, the impaired voltage–calcium coupling may lead to discordant interactions of membrane ion channels and intracellular calcium-handling proteins, which may evolve into arrhythmias.

### 4.2 Cardiac alternans serves as the electrophysiological substrate for ventricular arrhythmias of CIT

Prolonged APD and CaTD may lead to changed refractoriness in generating abnormal depolarization or intracellular calcium release, which are responsible for AP and CaT alternans (Liu et al., 2018; Qu and Weiss, 2023). Previous studies have indicated that SDA is inclined to generate lethal tachyarrhythmias (Cacheux et al., 2019; Liao et al., 2021). No SDA was found in all CIT-treated hearts nor in SAL hearts. However, after quantification of the CaT alternans ratio, we observed that hearts with the most severe arrhythmias would first express serious CaT alternans in a specific region, and the regions with electrical remodeling may provide the substrate for the initiation of arrhythmias under increased stress. Prolonged APD in spatially discordant regions caused increased APD dispersion; the AP repolarization in regions with short APD would finish at first and conduct to nearby regions, while the conduction would be blocked or deviated because of the not fully depolarized regions with prolonged APD, thus causing incomplete depolarization and formation of AP alternans. The AP alternans correlated well with CaT alternans. Moreover, being triggered by action potentials, the release of sarcoplasmic reticulum was well coordinated with APs, while the impaired Vm–Ca^2+^ coupling may interrupt the correlation and induce irregular calcium release and calcium alternans (Kanaporis et al., 2023). Combining the results of decreased I_Ca-L_ current and [Ca^2+^]_i_ after CIT treatment, the membrane potential may also be influenced, which may, in return, result in AP oscillation, AP alternans, and CaT alternans.

### 4.3 Integration of APD prolongation in cardiomyocytes and transcriptomics

In our study, we found that APD was prolonged in spatially discordant regions of ventricles in CIT-treated hearts by optical mapping results, and the transcriptomics data revealed that KCND2, KCNH2, KCNIP2, and KCNE1 were reduced following treatment with CIT, which are related to potassium channels and APD prolongation. Although there have been studies suggesting that CIT affects Nav1.5 in HEK293 (Nakatani and Amano, 2021), our findings from ECG (QRS wave morphology), optical mapping (conduction velocity), and transcriptomics data did not demonstrate any alterations in Nav1.5 expression or function. This absence of change may be attributed to compensatory mechanisms that arise following prolonged exposure to CIT. More research may be needed in future to explore the effects of CIT on Nav1.5 in different cell types or species, especially in human cardiomyocytes. In rodent hearts, fast transient outward K^+^ currents (I_to, f_) are generated by K_V_4 α-units (i.e., K_V_4.2 and K_V_4.3) and the Ca^2+^-sensing auxiliary subunit (KCNIP2), with KCND2 being the main gene that codes for K_V_4.2. Meanwhile, KCNE1 also plays a critical role in the regulation of K_V_4.3. We found that APD was profoundly prolonged during the entire repolarization process of CIT-treated cardiomyocytes, as shown by the whole-cell patch clamp technique, and I_to_ current density was remarkably reduced. Even though the protocol was used for total potassium current recording and peak current density calculated as I_to_, the variation tendency of I_to_ caused by CIT was confirmed. Prolonged APD_30_ was consistent with weakened I_to_, while APD_90_ was also prolonged at the same time, suggesting that I_ks_ and I_kr_ may be influenced to some extent since the mRNA expressions of KCNE1 and KCNH2 were also downregulated (KCNE1 is involved in I_ks,_ and KCNH2 is the main coding gene of I_kr_). Additionally, Chen et al. found that the polymorphisms of the KCNQ1, KCNE1, and KCNH2 genes have a complementary effect on escitalopram-induced QTc prolongation in elderly patients (Chen et al., 2024). Meanwhile, the Kv4.2 protein level was significantly reduced, which is the main component of I_to_. Taken together, CIT had a detrimental effect in prolonging APD by downregulating the activities of multiple potassium channels. Admittedly, the electrophysiology of rodent hearts cannot be completely equivalent to that of human cardiomyocytes or iPSCs because of the action potential plateau. However, experiments based on murine hearts can serve as a good example for studying arrhythmogenesis in drug-induced arrhythmias based on the repolarization and potassium current. Moreover, in our I_CaL_ current tests using the patch clamp, we also recorded a reduction in I_Ca-L_ current after CIT treatment. This might also be one of the reasons for the prolongation of APD.

### 4.4 Increased cardiac alternans and I_to_ downregulation in CIT-related QT prolongation and cardiac arrhythmias

Fluctuation of the sarcoplasmic reticulum calcium content plays a key role in cardiac alternans (Fakuade et al., 2021). Meanwhile, Qu et al. also mentioned that alterations in the expression or function of various calcium handling proteins are responsible for most cardiac alternans (Qu et al., 2013). In our study, we failed to observe any change in the expression of calcium-handling proteins in CIT-treated hearts, even though cardiac alternans are proven to not rely on calcium fluctuation (Picht et al., 2006); however, we found reduced [Ca^2+^]_i_ and I_Ca-L_ in the patch-clamp technique, which was one of the factors that generate CaT alternans. Meanwhile, to our knowledge, CaT alternans are widely accepted to precede AP alternans and are a predictor of arrhythmias (Kanaporis and Blatter, 2015). Although a considerable number of researchers still hold different opinions, they believe that prolonged action potentials and AP restitution play key roles in generation of AP alternans (Watanabe et al., 2012; Bingen et al., 2013; Kanaporis et al., 2023). CaT alternans and AP alternans are synchronized in time and magnitude (Kanaporis et al., 2023), and shortened APD rescued calcium alternans (Kanaporis et al., 2019). In addition, short APs generated by K^+^ channel activity have been associated with Vm-driven alternans (Fish and Antzelevitch, 2008). A recent study also demonstrated that activation of SK channels suppressed AP and CaT alternans (Kanaporis and Blatter, 2023). These studies suggest that potassium channels are widely linked to AP and CaT alternans. In our study, downregulation of I_to_ and other potential potassium currents prolonged APD and evoked aggravated AP alternans, which induced severe accompanying calcium alternans and led to ventricular arrhythmias. In addition, potassium current enhancement may alleviate AP alternans by weakening APD–CaT coupling (Livshitz and Rudy, 2007). I_to_ downregulation (potassium current weakening) then, in turn, enhanced AP alternans. APD–CaT uncoupling can also lead to CaT alternans in the absence of AP alternans (Kanaporis et al., 2023). The chaotic relationship of Vm and CaT may thus play an auxiliary role in the genesis of calcium alternans, even though we cannot currently clarify the complex and sophisticated interactions between the membrane channels and calcium-handling proteins. In general, CaT alternans manifested as the arrhythmogenic substrate after CIT treatment, and it was mainly generated by downregulation of Ito, weakened I_CaL_, and the impaired coupling of AP and CaT.

## 5 Conclusion

Regions with severe cardiac alternans associated with I_to_ downregulation contributed to citalopram-related reentrant ventricular arrhythmias. Potassium channel-related genes likely underlie the proarrhythmic mechanism and may be potential novel targets for preventing citalopram-related arrhythmias.

## Data Availability

The original contributions presented in the study are publicly available. This RNA-seq data can be found here: https://www.ncbi.nlm.nih.gov/bioproject/PRJNA1308822.

## References

[B1] Akturk G. Micili S. C. Gursoy Doruk O. Hocaoglu N. Akan P. Ergur B. U. (2023). Effects of nicorandil on QT prolongation and myocardial damage caused by citalopram in rats. Biotech. Histochem 98 (7), 479–491. 10.1080/10520295.2023.2233417 37466068

[B2] Aras K. Gams A. Faye N. R. Brennan J. Goldrick K. Li J. (2022). Electrophysiology and arrhythmogenesis in the human right ventricular outflow tract. Circ. Arrhythm. Electrophysiol. 15 (3), e010630. 10.1161/circep.121.010630 35238622 PMC9052172

[B3] Beach S. R. Celano C. M. Sugrue A. M. Adams C. Ackerman M. J. Noseworthy P. A. (2018). QT prolongation, torsades de pointes, and psychotropic medications: a 5-year update. Psychosomatics 59 (2), 105–122. 10.1016/j.psym.2017.10.009 29275963

[B4] Bersell K. R. Yang T. Mosley J. D. Glazer A. M. Hale A. T. Kryshtal D. O. (2023). Transcriptional dysregulation underlies both monogenic arrhythmia syndrome and common modifiers of cardiac repolarization. Circulation 147 (10), 824–840. 10.1161/CIRCULATIONAHA.122.062193 36524479 PMC9992308

[B5] Bingen B. O. Neshati Z. Askar S. F. Kazbanov I. V. Ypey D. L. Panfilov A. V. (2013). Atrium-specific Kir3.x determines inducibility, dynamics, and termination of fibrillation by regulating restitution-driven alternans. Circulation 128 (25), 2732–2744. 10.1161/circulationaha.113.005019 24065610

[B6] Cacheux M. Strauss B. Raad N. Ilkan Z. Hu J. Benard L. (2019). Cardiomyocyte-specific STIM1 (stromal interaction molecule 1) depletion in the adult heart promotes the development of arrhythmogenic discordant alternans. Circ. Arrhythm. Electrophysiol. 12 (11), e007382. 10.1161/circep.119.007382 31726860 PMC6867678

[B7] Chen Z. Xu Z. Gao C. Chen L. Tan T. Jiang W. (2024). Escitalopram-induced QTc prolongation and its relationship with KCNQ1, KCNE1, and KCNH2 gene polymorphisms. J. Affect Disord. 347, 399–405. 10.1016/j.jad.2023.11.084 38000475

[B8] Chien Y. S. Weng C. J. Wu S. J. Li C. H. Lin J. C. Huang J. L. (2023). Levosimendan attenuates electrical alternans and prevents ventricular arrhythmia during therapeutic hypothermia in isolated rabbit hearts. Heart Rhythm. 20 (5), 744–753. 10.1016/j.hrthm.2023.02.014 36804540

[B9] Cooke M. J. Waring W. S. (2013). Citalopram and cardiac toxicity. Eur. J. Clin. Pharmacol. 69 (4), 755–760. 10.1007/s00228-012-1408-1 22996077

[B10] Eslami A. Lujan J. (2010). Western blotting: sample preparation to detection. J. Vis. Exp. 44, 2359. 10.3791/2359 21189462 PMC3185633

[B11] Fakuade F. E. Steckmeister V. Seibertz F. Gronwald J. Kestel S. Menzel J. (2021). Altered atrial cytosolic calcium handling contributes to the development of postoperative atrial fibrillation. Cardiovasc Res. 117 (7), 1790–1801. 10.1093/cvr/cvaa162 32520995 PMC8208741

[B12] Faraj P. Størset E. Hole K. Smith G. Molden E. Dietrichs E. S. (2023). Pro-arrhythmic effect of escitalopram and citalopram at serum concentrations commonly observed in older patients - a study based on a cohort of 19,742 patients. EBioMedicine 95, 104779. 10.1016/j.ebiom.2023.104779 37639937 PMC10474154

[B13] Fish J. M. Antzelevitch C. (2008). Cellular mechanism and arrhythmogenic potential of T-wave alternans in the Brugada syndrome. J. Cardiovasc Electrophysiol. 19 (3), 301–308. 10.1111/j.1540-8167.2007.01025.x 18031511 PMC2367008

[B14] Frey A. Saxon V. M. Popp S. Lehmann M. Mathes D. Pachel C. (2016). Early citalopram treatment increases mortality due to left ventricular rupture in mice after myocardial infarction. J. Mol. Cell Cardiol. 98, 28–36. 10.1016/j.yjmcc.2016.07.002 27397875

[B15] Friberg L. E. Isbister G. K. Duffull S. B. (2006). Pharmacokinetic-pharmacodynamic modelling of QT interval prolongation following citalopram overdoses. Br. J. Clin. Pharmacol. 61 (2), 177–190. 10.1111/j.1365-2125.2005.02546.x 16433872 PMC1884996

[B16] Fukaya H. Plummer B. N. Piktel J. S. Wan X. Rosenbaum D. S. Laurita K. R. (2019). Arrhythmogenic cardiac alternans in heart failure is suppressed by late sodium current blockade by ranolazine. Heart Rhythm. 16 (2), 281–289. 10.1016/j.hrthm.2018.08.033 30193854

[B17] Handa B. S. Li X. Aras K. K. Qureshi N. A. Mann I. Chowdhury R. A. (2020). Granger causality–based analysis for classification of fibrillation mechanisms and localization of rotational drivers. Circ. Arrhythm. Electrophysiol. 13 (3), e008237. 10.1161/CIRCEP.119.008237 32064900 PMC7069398

[B18] Honig G. Jongsma M. E. van der Hart M. C. Tecott L. H. (2009). Chronic citalopram administration causes a sustained suppression of serotonin synthesis in the mouse forebrain. PLoS One 4 (8), e6797. 10.1371/journal.pone.0006797 19710918 PMC2728775

[B19] Kanaporis G. Blatter L. A. (2015). The mechanisms of calcium cycling and action potential dynamics in cardiac alternans. Circ. Res. 116 (5), 846–856. 10.1161/circresaha.116.305404 25532796 PMC4344847

[B20] Kanaporis G. Blatter L. A. (2023). Activation of small conductance Ca(2+) -activated K(+) channels suppresses Ca(2+) transient and action potential alternans in ventricular myocytes. J. Physiol. 601 (1), 51–67. 10.1113/jp283870 36426548 PMC9878619

[B21] Kanaporis G. Kalik Z. M. Blatter L. A. (2019). Action potential shortening rescues atrial calcium alternans. J. Physiol. 597 (3), 723–740. 10.1113/jp277188 30412286 PMC6355632

[B22] Kanaporis G. Martinez-Hernandez E. Blatter L. A. (2023). Calcium- and voltage-driven atrial alternans: insight from [Ca](i) and V(m) asynchrony. Physiol. Rep. 11 (10), e15703. 10.14814/phy2.15703 37226365 PMC10209431

[B23] Khwaounjoo P. Sands G. B. LeGrice I. J. Ramulgun G. Ashton J. L. Montgomery J. M. (2022). Multimodal imaging shows fibrosis architecture and action potential dispersion are predictors of arrhythmic risk in spontaneous hypertensive rats. J. Physiol. 600 (18), 4119–4135. 10.1113/jp282526 35984854 PMC9544618

[B24] Krøll J. Jespersen C. H. B. Kristensen S. L. Fosbøl E. L. Vinding N. E. Lippert F. (2022). Use of torsades de pointes risk drugs among patients with out-of-hospital cardiac arrest and likelihood of shockable rhythm and return of spontaneous circulation: a nationwide study. Resuscitation 179, 105–113. 10.1016/j.resuscitation.2022.08.008 35964772

[B25] Lei M. Huang C. L. (2020). Cardiac arrhythmogenesis: a tale of two clocks? Cardiovasc Res. 116 (14), e205–e209. 10.1093/cvr/cvz283 31800017 PMC7695354

[B26] Lei M. Wu L. Terrar D. A. Huang C. L. (2018). Modernized classification of cardiac antiarrhythmic drugs. Circulation 138 (17), 1879–1896. 10.1161/circulationaha.118.035455 30354657

[B27] Li P. Qin D. Chen T. Hou W. Song X. Yin S. (2023a). Dysregulated Rbfox2 produces aberrant splicing of Ca(V)1.2 calcium channel in diabetes-induced cardiac hypertrophy. Cardiovasc Diabetol. 22 (1), 168. 10.1186/s12933-023-01894-5 37415128 PMC10324275

[B28] Li Y. Yang J. Zhang R. Chen T. Zhang S. Zheng Y. (2023b). Dual-dye optical mapping of hearts from RyR2 R2474S knock-in mice of catecholaminergic polymorphic ventricular tachycardia. J. Vis. Exp. 202. 10.3791/65082 38189464

[B29] Liao J. Zhang S. Yang S. Lu Y. Lu K. Wu Y. (2021). Interleukin-6-mediated-Ca(2+) handling abnormalities contributes to atrial fibrillation in sterile pericarditis rats. Front. Immunol. 12, 758157. 10.3389/fimmu.2021.758157 34975847 PMC8716408

[B30] Liu W. Kim T. Y. Huang X. Liu M. B. Koren G. Choi B. R. (2018). Mechanisms linking T-wave alternans to spontaneous initiation of ventricular arrhythmias in rabbit models of long QT syndrome. J. Physiol. 596 (8), 1341–1355. 10.1113/jp275492 29377142 PMC5899976

[B31] Livak K. J. Schmittgen T. D. (2001). Analysis of relative gene expression data using real-time quantitative PCR and the 2(-Delta Delta C(T)) method. Methods 25 (4), 402–408. 10.1006/meth.2001.1262 11846609

[B32] Livshitz L. M. Rudy Y. (2007). Regulation of Ca2+ and electrical alternans in cardiac myocytes: role of CAMKII and repolarizing currents. Am. J. Physiol. Heart Circ. Physiol. 292 (6), H2854–H2866. 10.1152/ajpheart.01347.2006 17277017 PMC2274911

[B33] Nakatani Y. Amano T. (2021). Contributions of S- and R-citalopram to the citalopram-induced modulation of the function of Nav1.5 voltage-gated sodium channels. Eur. J. Pharmacol. 908, 174316. 10.1016/j.ejphar.2021.174316 34280395

[B34] Picht E. DeSantiago J. Blatter L. A. Bers D. M. (2006). Cardiac alternans do not rely on diastolic sarcoplasmic reticulum calcium content fluctuations. Circ. Res. 99 (7), 740–748. 10.1161/01.Res.0000244002.88813.91 16946134

[B35] Qu Z. Weiss J. N. (2023). Cardiac alternans: from bedside to bench and back. Circ. Res. 132 (1), 127–149. 10.1161/circresaha.122.321668 36603066 PMC9829045

[B36] Qu Z. Nivala M. Weiss J. N. (2013). Calcium alternans in cardiac myocytes: order from disorder. J. Mol. Cell Cardiol. 58, 100–109. 10.1016/j.yjmcc.2012.10.007 23104004 PMC3570622

[B37] Roden D. M. (2019). A current understanding of drug-induced QT prolongation and its implications for anticancer therapy. Cardiovasc. Res. 115 (5), 895–903. 10.1093/cvr/cvz013 30689740 PMC7967705

[B38] Rohrbacher J. Bex P. Gallacher D. J. (2012). Citalopram metabolites inhibit IKs and IKr differentially: is this a possible explanation for the sudden deaths in dogs? J. Pharmacol. Toxicol. Methods 66 (2), 170. 10.1016/j.vascn.2012.08.040

[B39] Ryan K. Benz P. Zosel A. Farkas A. Theobald J. (2022). QTc Prolongation in Poison Center Exposures to CredibleMeds List of Substances with “Known Risk of Torsades de Pointes”. Cardiovasc Toxicol. 22 (9), 866–877. 10.1007/s12012-022-09764-4 35930218

[B40] Tampi R. R. Balderas M. Carter K. V. Tampi D. J. Moca M. Knudsen A. (2015). Citalopram, QTc Prolongation, and Torsades de Pointes. Psychosomatics 56 (1), 36–43. 10.1016/j.psym.2014.09.002 25619672

[B41] Vieweg W. V. Hasnain M. Howland R. H. Hettema J. M. Kogut C. Wood M. A. (2012). Citalopram, QTc interval prolongation, and torsade de pointes. How should we apply the recent FDA ruling? Am. J. Med. 125 (9), 859–868. 10.1016/j.amjmed.2011.12.002 22748401

[B42] Wang L. Olivas A. Francis Stuart S. D. Tapa S. Blake M. R. Woodward W. R. (2020). Cardiac sympathetic nerve transdifferentiation reduces action potential heterogeneity after myocardial infarction. Am. J. Physiol. Heart Circ. Physiol. 318 (3), H558–H565. 10.1152/ajpheart.00412.2019 31975627 PMC7199228

[B43] Watanabe M. Yokoshiki H. Mitsuyama H. Mizukami K. Ono T. Tsutsui H. (2012). Conduction and refractory disorders in the diabetic atrium. Am. J. Physiol. Heart Circ. Physiol. 303 (1), H86–H95. 10.1152/ajpheart.00010.2012 22561303

[B44] Witchel H. J. Pabbathi V. K. Hofmann G. Paul A. A. Hancox J. C. (2002). Inhibitory actions of the selective serotonin re-uptake inhibitor citalopram on HERG and ventricular L-type calcium currents. FEBS Lett. 512 (1-3), 59–66. 10.1016/s0014-5793(01)03320-8 11852052

[B45] Yamazaki M. Tomii N. Tsuneyama K. Takanari H. Niwa R. Honjo H. (2022). Rotors anchored by refractory islands drive torsades de pointes in an experimental model of electrical storm. Heart Rhythm. 19 (2), 318–329. 10.1016/j.hrthm.2021.10.012 34678525 PMC8810573

[B46] Zhang Y. Li R. Jiang H. Hou Y. Zhang Y. Meng X. (2024). Salidroside modulates repolarization through stimulating Kv2.1 in rats. Eur. J. Pharmacol. 977, 176741. 10.1016/j.ejphar.2024.176741 38880221

[B47] Zhou X. Bueno-Orovio A. Orini M. Hanson B. Hayward M. Taggart P. (2016). In vivo and in silico investigation into mechanisms of frequency dependence of repolarization alternans in human ventricular cardiomyocytes. Circ. Res. 118 (2), 266–278. 10.1161/circresaha.115.307836 26602864 PMC4719495

