# Supplementary figures and images for: Abnormal Ca2+ handling and reduced Ito contribute to citalopram-induced QT prolongation and cardiac arrhythmias
Source: Front Pharmacol. 2025 Sep 4;16:1613438. doi: 10.3389/fphar.2025.1613438 (PMC12443570; doi:10.3389/fphar.2025.1613438)

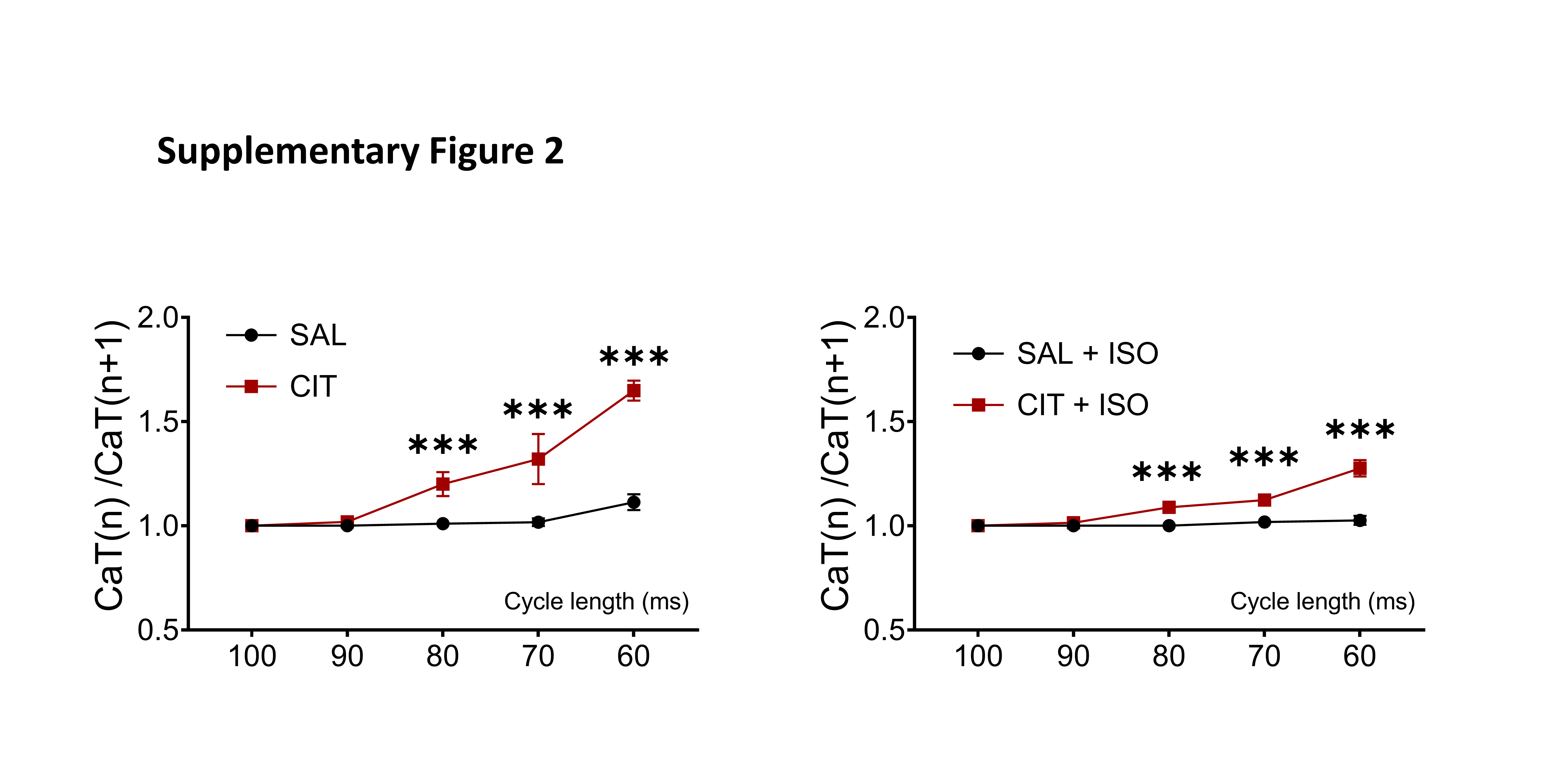

Supplement: Supplementary file 1 [file Image2.tif]

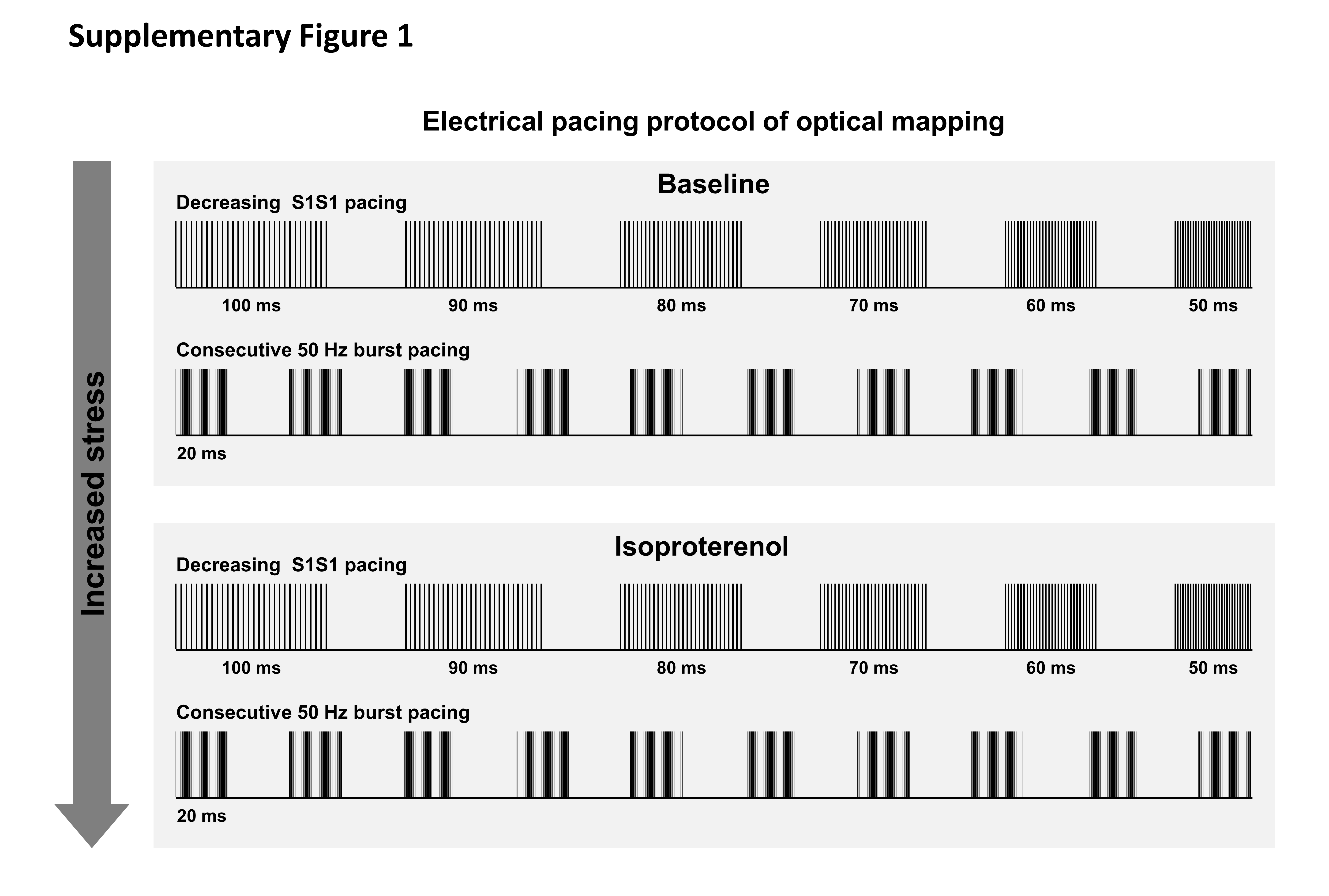

Supplement: Supplementary file 2 [file Image1.tif]
